# Identification and Molecular Characterization of Novel Mycoviruses in *Saccharomyces* and Non-*Saccharomyces* Yeasts of Oenological Interest

**DOI:** 10.3390/v14010052

**Published:** 2021-12-29

**Authors:** Dalila Crucitti, Marco Chiapello, Daniele Oliva, Marco Forgia, Massimo Turina, Francesco Carimi, Francesca La Bella, Davide Pacifico

**Affiliations:** 1 Dipartimento di Scienze Bio-Agroalimentari, Istituto di Bioscienze e BioRisorse (IBBR), C.N.R., Corso Calatafimi 414, 90129 Palermo, Italy; francesco.carimi@ibbr.cnr.it (F.C.); francesca.labella@ibbr.cnr.it (F.L.B.); 2 Dipartimento di Scienze Bio-Agroalimentari, Istituto per la Protezione Sostenibile delle Piante (IPSP), C.N.R., Strada delle Cacce, 73, 10135 Torino, Italy; marco.chiapello@ipsp.cnr.it (M.C.); marco.forgia@ipsp.cnr.it (M.F.); massimo.turina@ipsp.cnr.it (M.T.); 3 Istituto Regionale del Vino e dell’Olio (IRVO), Via Libertà 66, 90143 Palermo, Italy; daniele.oliva@regione.sicilia.it

**Keywords:** fungal viruses, totivirus, narnavirus, mitovirus, partitivirus, *Saccharomyces cerevisiae*, *Starmerella bacillaris*, RNAseq

## Abstract

Wine yeasts can be natural hosts for dsRNA, ssRNA viruses and retrotransposon elements. In this study, high-throughput RNA sequencing combined with bioinformatic analyses unveiled the virome associated to 16 *Saccharomyces cerevisiae* and 8 non-*Saccharomyces* strains of oenological interest. Results showed the presence of six viruses and two satellite dsRNAs from four different families, two of which—*Partitiviridae* and *Mitoviridae*—were not reported before in yeasts, as well as two ORFan contigs of viral origin. According to phylogenetic analysis, four new putative mycoviruses distributed in *Totivirus*, *Cryspovirus,* and *Mitovirus* genera were identified. The majority of commercial *S. cerevisiae* strains were confirmed to be the host for helper L-A type totiviruses and satellite M dsRNAs associated with the killer phenotype, both in single and mixed infections with L-BC totiviruses, and two viral sequences belonging to a new cryspovirus putative species discovered here for the first time. Moreover, single infection by a narnavirus 20S-related sequence was also found in one *S. cerevisiae* strain. Considering the non-*Saccharomyces* yeasts, *Starmerella bacillaris* hosted four RNAs of viral origin—two clustering in *Totivirus* and *Mitovirus* genera, and two ORFans with putative satellite behavior. This study confirmed the infection of wine yeasts by viruses associated with useful technological characteristics and demonstrated the presence of complex mixed infections with unpredictable biological effects.

## 1. Introduction

A wide diversity of mycoviruses has been reported in the major fungal taxa since the description of the first species in 1962 [1,2,3]. Fungal viruses lack an extracellular phase as free virions, and are transmitted intracellularly during cell division, cell fusion, and sporogenesis [1,3] with noticeable exceptions within the family *Genomoviridae* [4,5]. Mycovirus infections are usually cryptic or associated with mild symptoms, but in some cases, they can induce growth alteration, reduce the virulence, or kill other individuals of their host species [1,6,7].

The over 500 fungal viruses reported in the literature are characterized by simple or multi-segmented genomes which are packaged in isometric, spherical, bacilliform, or filamentous particles [8,9,10]. The majority of fungal viruses have double-stranded RNA (dsRNA) or positive single-stranded RNA (+ssRNA) genomes, although some of them have linear negative (-ssRNA) as well as circular ssDNA genomes [1]. According to the International Committee on Taxonomy of Viruses—ICTV (https://talk.ictvonline.org/taxonomy/; 2020 release, accessed on 16 December 2021)—mycoviruses with dsRNA genomes are grouped in seven families—*Chrysoviridae*, *Endornaviridae*, *Megabirnaviridae*, *Partitiviridae*, *Quadriviridae*, *Reoviridae*, *Totiviridae*, one genus *Botybirnavirus* in the kingdom *Orthornavirae*, and four recently established families—*Amalgaviridae*, *Curvulaviridae*, and *Picobirnaviridae* and *Polymycoviridae*. Mycoviruses with +ssRNA genomes are classified into eight families—*Alphaflexiviridae*, *Barnaviridae*, *Endornaviridae*, *Gammaflexiviridae*, *Hypoviridae*, *Narnaviridae*, *Mitoviridae*, and *Fusariviridae*. *Mymonaviridae* and *Metaviridae* families include mycoviruses with ssRNA and +ssRNA-RT genomes, respectively, while the family *Genomoviridae* was recently established for mycoviruses with a single-stranded DNA (ssDNA) genome [4]. Recently, a member of the *Rhabdoviridae* family with –ssRNA was identified for the first time in a fungal species [11]. Thanks to the improvement of cultivation-independent methods in virome research, new families are constantly proposed as the number of new species arises [8,11,12,13].

Several techniques can be applied for detecting the presence of mycoviruses such as virus purifications and electron microscopy, dsRNA extraction and cDNA cloning, total and small RNA sequencing through NGS techniques, rolling circle amplification (RCA), and total DNA sequencing [14,15]. Next-generation sequencing (NGS) analysis on host transcriptome [16,17,18] or small RNA (sRNA) libraries [19,20,21] allowed for the detection of new mycoviruses and new orphan open reading frames (ORFans) with no sequence similarity to proteins of other genomes [22], and to increase our knowledge about their diversity [2,15,23], evolution, and horizontal transfer between different hosts [24,25,26].

RNA elements found in yeasts of the *Saccharomyces sensu strictu* complex include three types of dsRNA viruses (L-A, L-BC, and M; family *Totiviridae*), three of ssRNA viruses (20S, 23S, and I329; family *Narnaviridae*), and five types of retrotransposons (Ty1–Ty5) [27,28]. These viruses can be found in single or mixed infections both in laboratory and wild strains [29,30,31]. *Totiviridae* is the most widespread family among the *Saccharomyces* yeasts, and in the type species *S. cerevisiae*, it is traditionally represented by four genotypes of Saccharomyces cerevisiae virus L-A (ScV-L-A1, ScV-L-A2 and ScV-L-A-lus, ScV-L-A28), three of S. cerevisiae L-BC (ScV-L-BC2, ScV-L-BC-La, and ScV-L-BC-lus), and four satellite M dsRNAs (ScV-M1, ScV-M2, ScV-M28, and ScV-Mlus) [32]. In the cytoplasm, dsRNA L-A acts as a helper virus for dsRNA M replication and encapsidation, while L-BC shows no helper activity [33]. The M dsRNAs encode four types of killer toxins (K1, K2, K28, and Klus, respectively) that are lethal to sensitive strains of the same or related yeast species [33]. Considering the killer behavior, yeast strains may show one of three phenotypes—killer (K), sensitive (S), or neutral (N). During the early phases of must fermentation, K strains secrete killer toxins that can be lethal to non-killer S strains, causing severely stuck fermentations, particularly when yeast starter cultures are suppressed by wild-type K strains of the grapes [34]. N strains are not killed by the killer, nor do they kill the sensitive ones [35]. Killer toxins are widespread across different yeast taxonomic groups and habitats, providing benefits in terms of direct competitions among strains and possibly ecological interactions, including predation, protection from pathogens, habitat colonization [36]. The list of dsRNA-encoded killer toxins with antifungal activity is constantly enriched as new studies on the *Saccharomyces* complex are carried on [37,38]. Viruses of family *Totiviridae* were found also in other species within the *Saccharomyces* complex such as *Saccharomyces kudriavzevii* [27,31], *S. paradoxus* [31], and *S. uvarum* [39,40].

The presence of ssRNAs in *S. cerevisiae* has been known since 1971 [41]. Saccharomyces cerevisiae 20S narnavirus (ScNV-20S) and S. cerevisiae 23S narnavirus (ScNV-23S) were for a long time the only known members belonging to the *Narnaviridae* family detected in yeasts of the *Saccharomyces* complex [42]. Recently, Mardanov [29] identified a third narnavirus ssRNA in *S. cerevisiae* strain I-329 clustering in a well-separated phylogenetic group together with ScNV-20S.

Double-strand RNA elements found in non-*Saccharomyces* yeast of oenological interest include only members of the family *Totiviridae* involved in the killer phenotype of the host [43]. L-A and M dsRNA types were characterized in *Hanseniaspora uvarum* and *Zygosaccharomyces bailii* [44] and *Torulaspora delbrueckii* [45].

Budding yeast is also a model eukaryotic host for the virus infection of plant and animal viruses [46], providing numerous insights in the genetics of the host factors involved in virus replication [47]. In this study, we applied RNAseq analysis to verify the presence of mycoviruses in both *Saccharomyces* and non-*Saccharomyces* isolates of oenological interest. Our aim was to lay the bases for future studies assessing the effect of the virus/yeast interaction with possible new virus types found through NGS approaches, as well as to identify viruses in new yeast isolates with technological characteristics useful for the production of high-quality wines.

## 2. Materials and Methods

### 2.1. Yeast Isolate Maintenance

Twenty-four yeast strains of oenological interest were investigated in this study (Table 1). Among them, 16 were *S. cerevisiae* strains used as commercial starters in oenology that were collected from packages of active dry yeasts purchased on the market. The remaining eight non-*Saccharomyces* species came from the collections of the Regional Institute of Wine and Oil (IRVO, Palermo, Italy), or of the Department of Plant Biology of the University of Perugia (DBVPG, Perugia, Italy). Cryogenically preserved (−80 °C) strains were first cultured on WL Nutrient Agar (Yeast Extract 4.0 g/L, Enzymatic Digest of Casein 5.0 g/L, Dextrose 50.0 g/L, Monopotassium Phosphate 0.55 g/L, Potassium Chloride 0.425 g/L, Calcium Chloride 0.125 g/L, Magnesium Sulfate 0.125 g/L, Ferric Chloride 0.0025 g/L, Manganese Sulfate 0.0025 g/L, Bromocresol Green 0.022 g/L, Agar 20.0 g/L) and then maintained at 4 °C as slant tubes in YPD Agar (Yeast Extract 10 g/L, Peptone 20 g/L, Dextrose 20 g/L, Agar 20 g/L) or in Malt Agar (Malt Extract 30 g/L, Agar 15 g/L). Cultures of each strain were prepared in YPD medium (Yeast Extract 10 g/L, Peptone 20 g/L, Dextrose 20 g/L) and incubated under shaking at 37 °C for 24–48 h. Four milliliters of cellular suspensions were centrifuged at 4100 rpm for 20 min at 4 °C; the obtained pellet was lyophilized and stored at −80 °C until RNA extraction.

### 2.2. Total RNA Extraction and RNAseq

Total RNA was extracted from yeast pellets using the E.Z.N.A. Fungal RNA Mini Kit (Omega Bio-tek, Norcross, GA, USA) and resuspended in 30 μL of RNase-free water. Nucleic acid extracts were analyzed in a NanoDrop 2000 Spectrophotometer (Thermoscientific, Waltham, MA, USA) to evaluate their concentration and estimate their purity, and then stored at −80 °C. A total of 24 RNA samples were pooled by mixing 1 μg of RNA from each fungal sample to sequence more than one isolate per library. Eight μg total RNA were sent to sequencing facilities of Macrogen (Seoul, Korea) who performed the ribosomal RNA (rRNA) depletion (Ribo-ZeroTM Gold Kit, Epicentre, Madison, WI, USA), the cDNA library building (TrueSeq total RNA sample kit, Illumina, San Diego, CA, USA), and sequencing by an Illumina HiSeq4000 system generating paired-end sequences.

### 2.3. Transcriptome Assembly

Bioinformatics analysis of RNAseq raw reads was performed as previously detailed [48,49]. Briefly, de novo assembly was made with Trinity (2.3.2) [50], and assembled contigs were aligned with BLAST (ver. 2.6) [51] to search for homology with viral sequences against a custom viral proteins database. Bowtie2 (2.2.9) [52] and SAMtools (0.1.19) [53] were used to map reads on assembled contigs and visualized with IGV [54]; open reading frames were searched with ORF finder (https://www.ncbi.nlm.nih.gov/orffinder/, accessed on 21 July 2021).

### 2.4. ORFan Contig Detection

The assembled contigs were blasted, using DIAMOND (0.9.26), against the NCBI non-redundant whole database (version–October 2019). All hits were discarded, while the unmatched contigs with nucleic size over 1 kb and encoding a protein of at least 150 amino acids (~15 kDa) were kept. The latter were used to map reads considering their orientation, i.e., whether they mapped in sense or antisense orientation. Contigs with only positive or negative mapped reads were discarded, while the remaining contigs formed the ORFans pool [11].

### 2.5. Virus Detection in Specific Isolates

The distribution of the in silico-assembled viruses was verified for each of the 24 yeast isolates by reverse transcription followed by real-time PCR. To this aim, total RNA extracts were used as a template for cDNA synthesis using the High-Capacity cDNA Reverse Transcription Kit (Thermo Fisher scientific, Waltham, MA, USA) following manufacturer’s instructions. Real-time PCR reactions were carried out in a CFX Connect real-time PCR Detection System (Bio-Rad Laboratories, Hercules, CA, USA), adding 2 μL of 1:10 cDNA dilutions (in sterile distilled water, SDW) to 20 μL real-time PCR mix containing iTaq Universal SYBR Green Supermix, 200 nM (each) primers and SDW. A melting curve analysis was performed to check for unspecific real-time PCR products. In order to confirm the sequence of the assembled contigs, fragments of viral genomes were amplified by PCR reactions on the cDNA using specific primers designed in this study. All primers used in this work are reported in the Supplementary Table S1. Purified PCR products were selected and sent to the Eurofins Genomics sequencing service (Eurofins Genomics, Ebersberg, Bayern, Germany). All sequences determined in this work were submitted to the NBCI, and accession numbers were assigned (Table 2).

### 2.6. ORFan Detection in Specific Isolates and DNA Integration Assay

In silico detected ORFan contigs were searched in the twenty-four isolates studied through real-time PCR using the same procedure described for viral detection. Real-time PCR primers targeting the specific ORFan contigs are reported in Supplementary Table S1. For each yeast isolate, real-time PCR reactions were performed on cDNA and on total nucleic acids to verify if DNA corresponding to the ORFan coding sequence is present, therefore confirming (or excluding) the exclusive RNA nature of the ORFan, thus excluding (or confirming) an integration in the host genome or a DNA intermediate during replication.

### 2.7. ORF Prediction and Phylogenetic Analyses

ORF predictions were performed using the ORF finder tool from NCBI, and predictions were made selecting the “standard” genetic code for all viral contigs, except the one closely related to mitoviruses, generally hosted in the mitochondria. The putative function of the predicted protein was established by BLASTp analysis, looking at the function of the closest proteins in the NCBI database. The predicted protein sequences were analyzed through a BLASTp search using the domain finder option to evaluate the presence of any conserved domain in the sequence (such as the viral polymerase GDD conserved domain).

To assess the genomic variability of the totivirus isolates associated to the killer phenotype, ScV-L-A and ScV-L-BC CPs as well as of the ScV-M dsRNAs, nucleotide sequences were trimmed, aligned by CLUSTALW implemented in the MEGA X software [55] and compared to sequences of isolates retrieved from the GenBank (Supplementary Table S2). Phylogenetic trees were obtained using the Maximum likelihood method with the Kimura 2-parameter model and 1000 bootstrap replicates.

For the remaining phylogenetic analyses, multiple sequence alignments of viral RdRp amino acid sequences were performed with CLUSTAL Omega, and gaps were removed using trimAl v1.3 (strict mode) [56]. Maximum likelihood phylogenetic trees were constructed using the RtREV with Freqs. (+F) model and the Gamma Distributed with Invariant Sites (G+I) implemented in the MEGA X software. Bootstrap values are from 1000 replicates. Percentage identity values were obtained using the p-distance method of MEGA X, which automatically calculates the distances for nucleotide and amino acid sequences.

## 3. Results

A single sequencing run of the 24 pooled yeast isolates produced 135,345,688 total reads deposited in Sequence Read Archive (SRA) on the NCBI server (Bioproject PRJNA768663). Following the Trinity run, a total of 25,321 contigs were obtained. A BLAST search of a custom prepared viral database identified 13 putative viral contigs. Among those, seven contigs contained almost complete viral genomes; three contigs encoded viral RdRp, one a CP sequence, and two contigs viral toxins. In all, our bioinformatics pipeline unveiled the presence of six distinct mycoviruses and two satellite dsRNAs included in *Mitoviridae*, *Narnaviridae*, *Partitiviridae*, and *Totiviridae* families. Reverse transcription followed by real-time PCR assays confirmed the presence of viral infections in 15 out 24 tested isolates, with 13/15 mixed infections (Table 3).

### 3.1. Totivirus Related Sequences

From the RNAseq assembly, we identified two dsRNA viral species, ScV-L-A and ScV-L-BC, included in the genus *Totivirus* (family *Totiviridae*) and one satellite dsRNA, ScV-M (Table 3).

The presence of ScV-L-A was revealed by a contig of 3456 nucleotides showing 99% identity with the reference L-A-2 strain 8F-13 (GenBank KC677754), encoding the putative viral RdRp (partial) and the CP (complete) ORFs. ScV-L-A infection was confirmed in 12 yeast isolates, and the presence of different genetic variants, two L-A-lus and ten L-A-2, was verified by sequence alignment and the phylogenetic analysis of a 647 bp-long fragment of the CP gene (Supplementary Figure S1). According to pairwise analysis, ScV-L-A CP sequences shared among them from 77.6% to 100% and from 95.3% to 100% of nucleotide and amino acid identity, respectively (Supplementary Table S3). Further analysis of the CP partial sequence revealed that the two isolates LA-lus-D254 and LA-lus-U43 shared 85.3% and 97.2% of nucleotide and amino acid identity between them, and values higher than 84.9% and 96.7% of nucleotide and amino acid identity with the reference L-A-lus sequence (GenBank JN819511), respectively. ScV-L-A isolates clustering in the L-A-2 phylogenetic group shared nucleotide and amino acid identities >99% among them as well as with the L-A-2 reference strain 8F-13 (Supplementary Table S3). In particular, the isolates LA2-L2226 showed 100% amino acid identity with LA2-2323, LA2-VL1, and LA2-FX10, as well as with the reference L-A-2 sequence. The isolates LA2-EC1118, LA2-QA23, LA2-X5, LA2-VR5, LA2-PDM, and LA2-EZ44 shared 100% amino acid identity among them, and 99.5% with the L-A-2 strain 8F-13.

Two contigs of 1165 and 308 bp corresponded to ScV-M-2 and ScV-M-lus satellite dsRNAs encoding the complete K-2 and the partial (73 aa) K-lus killer toxins, respectively. ScV-M dsRNAs were detected in 12 out 24 *S. cerevisiae* strains; among them, 11 strains carried the M-2 type and one strain the M-lus type (Table 3).

Following pairwise analysis, ScV-M-2 sequences obtained in this work shared among them from 98.6% to 100% and from 97.5% to 100% of nucleotide and amino acid identity, respectively (Supplementary Table S4). A Further pairwise comparison including ScV-M-2 sequences from the GenBank showed nucleotide and amino acid identity values ranging from 98.2–100% and 71.8–100%, respectively (Supplementary Table S4). Considering the overall structure of K2 preprotoxins, the presence of a conserved hydrophobic amino terminal portion (aa 27–45), three potential N-glycosylation sites (aa 177–180, 214–217, 261–264), and two cleavage sites (KR) recognized by the KEX1 (aa 220–221) and KEX2 (aa 267–268) proteases, was verified in all isolates except M2-FX10. In this latter case, we found a substitution of lysine with glutamic acid at position 220 at the first KR site. Moreover, we found additional amino acid substitutions in eight different positions of the K2 preprotoxins from different isolates as reported in Supplementary Figure S2.

The comparison of a 292 bp fragment located at the 5′ of M-lus RNA from Lalvin ICV D254 showed 97% nucleotide identity with the corresponding region of the reference strain Mlus-4 (GenBank GU723494). Amino acid sequence analysis predicted a defective ORF due to the presence of a stop codon at position 232–234 that was confirmed not only in the in-silico assembly, but also through Sanger sequencing of a PCR fragment obtained by RT-PCR.

Infection with ScV-L-BC was revealed by four contigs, one of 4211 bp and three of 4601 bp, sharing from 94.4% to 99.5% nucleotide identity with the corresponding genomic regions of the reference strain ScV-L-BC-2 (GenBank KX906605). All four contigs contained complete ORFs encoding RdRp and CP proteins. Eight out of the 24 tested yeasts were positive for ScV-L-BC infection. According to the sequence analysis of an 801 bp-long fragment covering partial RdRp and CP genes, two commercial *S. cerevisiae* strains carried the ScV-L-BC-La type and six strains the ScV-L-BC-2 type (Table 3). Considering pairwise distances, ScV-L-BC sequences shared among them from 95.5% to 100% and from 97.4% to 100% of nucleotide and amino acid identity, respectively (Supplementary Table S5). The two ScV-L-BC isolates clustering in the LBC-La phylogenetic group (Supplementary Figure S3) shared 100% of nucleotide and amino acid identity among them, and 96.1% of nucleotide and 97% of amino acid identity with the reference L-BC-La sequence (GenBank: U01060) (Supplementary Table S5). The highest identity values were obtained among ScV-L-BC-2 type variants, sharing from 99.8% to 100% nucleotide and 100% amino acid identity, both among them and with the reference strain ScV-L-BC-2 (GenBank KX906605) (Supplementary Table S5).

**Figure 1:**
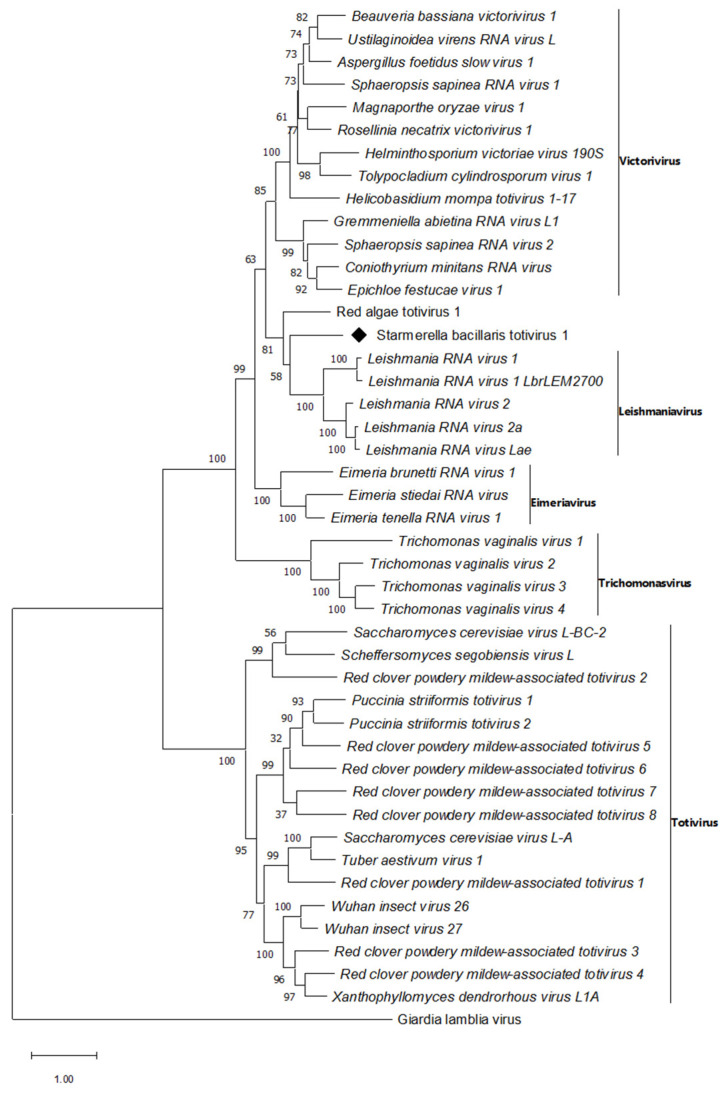
Phylogenetic analysis of Starmerella bacillaris totivirus 1 isolate Cz12 (SbTV1-Cz12). Maximum-likelihood tree was obtained following the alignment of RdRp amino acid sequences. Bootstrap values reported at the nodes are from 1000 replicates. The sequence determined in this work is indicated by ♦.

A contig of 5878 bp corresponding to a previously unknown virus within the family *Totiviridae* was identified by our bioinformatic pipeline and detected by real-time PCR on the cDNA obtained from *S. bacillaris* Cz12 total RNA (Table 3). A similarity search by BLASTp with the predicted amino acid sequence as query showed the maximum identity score with Red algae totivirus 1 Chiba7 (RaTV1, GenBank LC521327) from the red algae holobiont [55]; according to this result, the sequence was named Starmerella bacillaris totivirus 1 (SbTV1). A 5654 bp-long fragment of the SbTV1 genome was verified by Sanger sequencing of overlapping RT-PCR amplified segments with specific primers designed in this study (Supplementary Table S1). The sequence contained two distinct ORFs of 2649 bp (ORF2) and 2829 bp (ORF1) encoding the complete viral RdRp (882 aa) and CP (942 aa), respectively. SbTV1 showed the maximum CP amino acid identity value (40.3%) with Magnaporthe oryzae virus 2 (MoV2, GenBank BCL64969), while the RdRp highest identity value (43.9%) was obtained with RaTV1 (GenBank BBZ90082), respectively (Supplementary Tables S6 and S7). An overlap region (AUGA; nt 3166 to 3169) for the ribosomal slippage was found between the CP and RdRp ORFs. A phylogenetic analysis of complete RdRp amino acid sequences grouped SbTV1 and RaTV1 in a separated cluster within the *Totiviridae* family, confirming their close relationship (Figure 1).

### 3.2. Partitivirus Related Sequences

**Figure 2:**
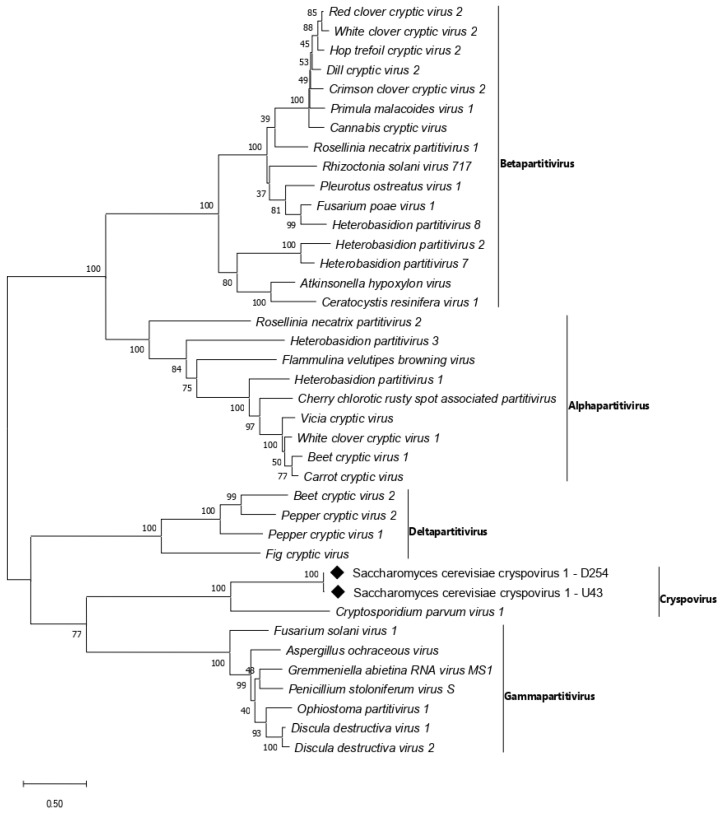
Phylogenetic analysis of Saccharomyces cerevisiae cryspovirus 1 isolates D254 (ScCV1-D254) and Saccharomyces cerevisiae cryspovirus 1 isolate U43 (ScCV1-U43). Maximum-likelihood tree was constructed based on the alignment of RdRp amino acid sequences. Bootstrap values reported at the nodes are from 1000 replicates. Sequences determined in this work are indicated by ♦.

Two contigs of 1689 bp and 1321 bp corresponding to RdRp (RNA1) and CP (RNA2) of an unknown virus within the family *Partitiviridae* were identified among the viral proteins database. A BLASTp search revealed the highest amino acid identity with Cryptosporidium parvum virus 1 (CSpV1, GenBank AAC47805). Reverse transcription followed by real-time PCR showed that two *S. cerevisiae* strains, ICV D254 and UVAFERM43, hosted both the RNA1 and the RNA2 of the putative partitivirus (Table 3). Fragments of 1552 bp and 1047 bp encoding the complete RdRp and CP ORFs were sequenced for both isolates, using specific primers designed in this study (Supplementary Table S1). Among them, D254 and U43 sequences showed 97.7% nucleotide and 99.4% amino acid identity values for the RdRp, and 98.9% nucleotide and 98.7% amino acid identity values for the CP, respectively. A pairwise comparison of D254 and U43 RdRp and CP complete sequences with all CSpV1 isolates from the GenBank showed the highest identity values (37% and 21.8%) with Cryptosporidium parvum virus 1 (GenBank BAU19319) RdRp and Cryptosporidium meleagridis virus CP (GenBank ABB02501), respectively (Supplementary Tables S8 and S9). A phylogenetic analysis of partial D254 and U43 RdRp (515 aa) sequences confirmed their position with members of the *Cryspovirus* cluster of *Partitiviridae* (Figure 2). According to these results, the two sequences were proposed as members of a new putative species provisionally named Saccharomyces cerevisiae cryspovirus 1 (ScCV1).

### 3.3. Narnavirus Related Sequences

**Figure 3:**
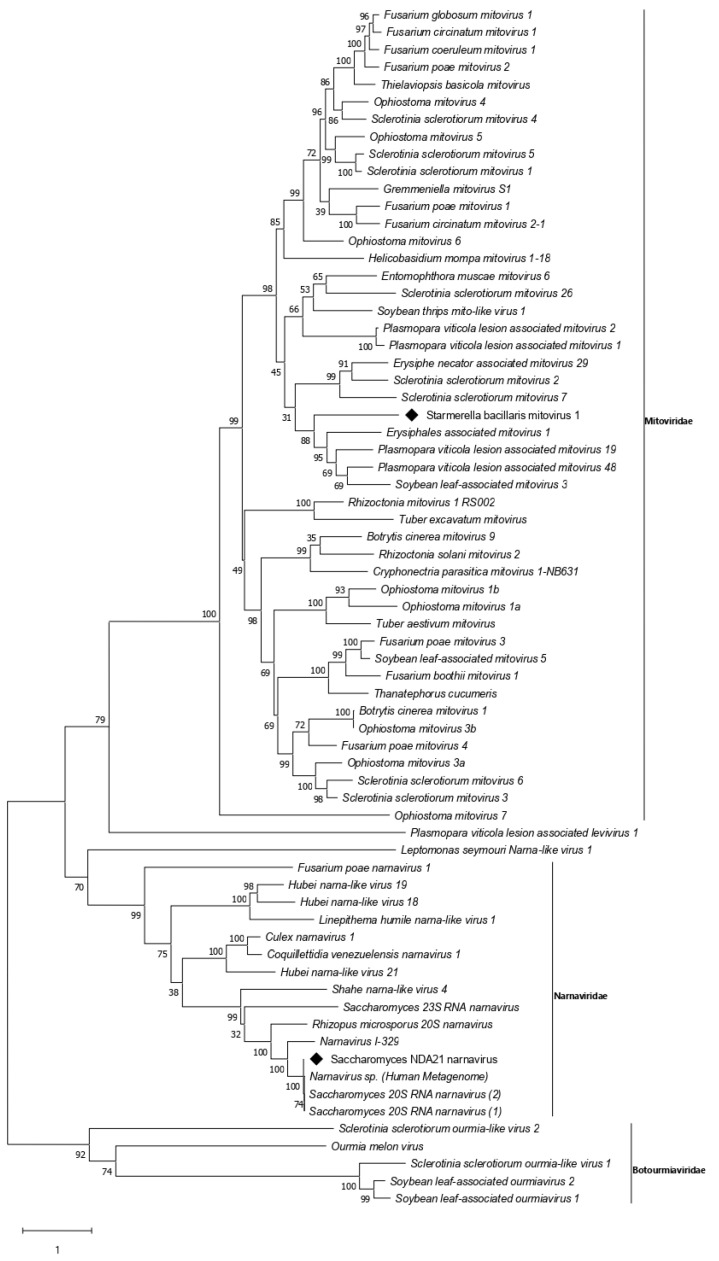
Phylogenetic analysis of Saccharomyces cerevisiae narnavirus NDA21 (ScNV-NDA21) and Starmerella bacillaris mitovirus 1 (SbMV1-Cz12). Maximum-likelihood tree was constructed based on the alignment of RdRp amino acid sequences. Bootstrap values reported at the nodes are from 1000 replicates. Sequences determined in this work are indicated by ♦.

The presence of a narnavirus in the pooled RNA was revealed by a contig of 2495 nt sharing 94.75% nucleotide identity with the *S. cerevisiae* narnavirus 20S RNA W (ScNV-20S, GenBank AF039063). The diagnostic screening showed that only *S. cerevisiae* NDA21 hosted this narnavirus. A comparison of the NV-NDA21 complete RdRp sequence amplified with specific primers showed amino acid identities values of 98.6% and 98.7% with the two ScNV 20S sequences available in the GenBank (AAA47824 and NP_660178), 55.9% with ScNV-I329 (GenBank QFU28543), and 30% with ScNV 23S (GenBank NP_660177) (Supplementary Table S10). A maximum likelihood phylogenetic analysis of RdRp (829 aa) confirmed its position in the ScNV-20S clade (Figure 3).

### 3.4. Mitovirus Related Sequences

**Figure 4:**
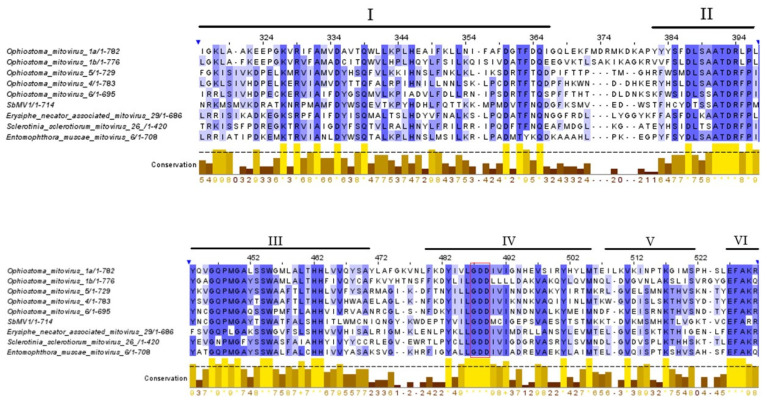
Comparison of amino acid sequence motifs in the RdRp-like proteins encoded by mitochondrial viruses and the proposed novel species Starmerella bacillaris mitovirus 1 (SbMV1). The alignment was made with the CLUSTAL Omega program. Conserved motifs (from I to VI) are indicated by lines above the sequence alignment. Identical aa residues are color-highlighted with blue; conserved and semi-conserved amino acid residues are color-highlighted with lilac and light lilac, respectively. The hallmark viral polymerase GDD tripeptide conserved domain is indicated by the red rectangle.

From the RNAseq assembly, we identified a 2465 nt-long contig encoding for one ORF producing the complete viral RdRp (714 aa) of a putative new mitovirus. The predicted protein alignment and phylogenetic analysis confirmed this sequence as a member of the genus *Mitovirus*, showing the highest amino acid sequence identity (35.97%) with the RdRp of Sclerotinia sclerotiorum mitovirus 26 (GenBank AWY10984). Reverse transcription followed by real-time PCR attributed this sequence to the *S. bacillaris* Cz12 isolate (Table 3). A 2297 bp-long fragment containing the complete RdRp ORF was verified following amplification and sequencing with specific primers designed on the contig (Supplementary Table S1). A BLASTp search showed that the RdRp amino acid sequence of the Cz12 mitovirus is most closely related to those of Sclerotinia sclerotiorum mitovirus 26 (SsMV26; GenBank AWY10984.1), Entomophthora muscae mitovirus 6 (EmMV6; GenBank QCF24450.1), and Erysiphe necator associated mitovirus 29 (EnMV29; GenBank QKI79977.1), with aa identities of 35.97%, 35.74%, and 34.56%, respectively. Sequence analysis evidenced the conserved domain Mitovir RNA pol at position 184–468 and confirmed the presence of the hallmark viral polymerase GDD tripeptide conserved domain, as well as of the six conserved motifs (I–VI) typical of the RdRps of mitochondrial viruses (Figure 4). The Maximum likelihood phylogenetic tree based on the RdRp amino acid sequence (714 aa) confirmed Cz12 within the clade III of mitoviruses [57] (Figure 3). According to these results, the provisional name Starmerella bacillaris mitovirus 1 (SbMV1) was given to this virus.

### 3.5. ORFan Sequences

**Figure 5:**
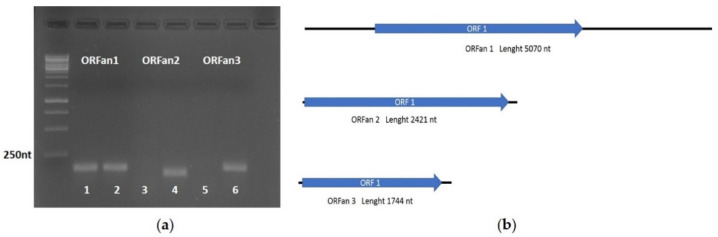
(**a**) Electrophoresis gel of the real-time PCR amplification products from ORFan1, ORFan2, and ORFan3 cDNAs. To confirm the RNA nature of the contigs, real-time PCR reaction was performed on cDNA samples and total nucleic acid samples from *Candida stellata* strain DBVPG 6714 and *Starmerella bacillaris* Cz12. 1: Total nucleic acid of *Candida stellata* DBVPG 6714; 2: cDNA of *Candida stellata* DBVPG 6714; 3 and 5: Total Nucleic of *Starmerella bacillaris* Cz12; 4 and 6: cDNA of *Starmerella bacillaris* Cz12; (**b**) Genome organization of the three ORFan contigs considered in our study. A black line represents the contig, while a blue arrow represents the open reading frame (ORF).

Our in-silico approach allowed the identification of eighteen contigs encoding for proteins without homology to known peptides deposited on available databases (ORFans). From these, the proportion of reads mapping on the positive or negative sense of each contig was screened manually, and only the contigs showing at least around 1/10 of the negative/positive reads proportion were kept for further analysis. The three resulting contigs that passed the last step were called ORFan1 to ORFan3, and information about their nucleotide sequences (sequence length, amino-acid length of the putative protein encoded, and the corresponding translated ORF) is displayed in Supplementary online Text S1. To detect ORFan1, 2, and 3 presence in the isolates from the collection and to confirm their viral nature as the RNA sequence not integrated in the host genome, real-time PCR amplifications on both total nucleic acids and the corresponding cDNAs were performed. Results obtained allowed for the identification of ORFan 2 and ORFan 3 in *Starmerella bacillaris* strain Cz12, confirming the absence of a specific signal when performing the PCR reaction on the total nucleic acid, therefore confirming that these segments do not have a corresponding DNA, and derive by RNA-dependent replication. However, ORFan1 was detected in *Candida stellata* strain DBVPG 6714, but the signal related to this contig was observed also in the total nucleic acid control, suggesting the genomic origin of ORFan1. Real time PCR products obtained using as a template the cDNA from yeast total RNA were separated through agarose gel electrophoresis (Figure 5a). Taken together, these results show that we were able to identify two ORFan sequences of putative viral origin (ORFan2 and ORFan3) that are found in the host isolate only as the RNA molecule and one sequence (ORFan1) that is found both as RNA and DNA. ORF distribution within the three ORFans is reported in Figure 5b.

## 4. Discussion

Some yeast species more than others are used in the fermentation of musts for their oenological qualities; nevertheless, the wine industry is constantly looking for new superior strains able to improve the quality of final products [58,59]. In the last fifteen years, many scientific works demonstrated the possibility of selecting non-*Saccharomyces* yeasts capable of improving the quality of wines produced by controlled mixed fermentation, where the inoculation is carried out with two different strains, a non-*Saccharomyces* and a *Saccharomyces*, usually sequentially [60]. The interest for the use of non-*Saccharomyces* yeasts in oenology is increasing, because they are able to decrease the alcohol content of wines, to increase the final concentration of glycerol and aromatic compounds, to produce proteolytic and pectinolytic activities as well as to influence the concentration of polysaccharides [58,61].

Yeasts are popular model organisms for virus research because they can be hosts for plant, animal, and human viruses [28]. Yeast-virus interactions underlie important mechanisms such as the killer system with several potential applications, including the production of food and beverages, biological control of plant pathogens, biocontrol of postharvest fungal alterations [62,63]. It is believed that the loss of anti-viral silencing defense genes in some budding yeast species is linked to the fitness gain obtained by hosting viruses supporting killer toxin replication [64]. Despite their biotechnological potential, the impact of yeast natural infections has been deeply investigated only for the killer system in species of the *Saccharomycetaceae* family and in few species of non*-Saccharomyces* yeasts employed for winemaking [43,65,66]. In this work, we searched for new viruses possibly able to affect the technological characteristics of both non-*Saccharomyces* and *Saccharomyces* strains using a high-throughput RNAseq approach combined to reverse transcription and real-time PCR, which were confirmed here to be suitable methods to unravel the presence of new viral species even in unicellular fungi [29]. The main advantage of using real-time PCR for amplification of reverse transcription products relies on the possibility of collecting data during the reaction exponential phase, when the quantity of the PCR product is directly proportional to the amount of template viral cDNA, thus providing information on the virus titer. Furthermore, a combined melting analysis allows evaluating accurately the reaction specificity [67].

With the exception of the putative viral ORFan sequences, the viral species found in the strains analyzed belong to virus families already reported in yeast [27] or in other unicellular organisms [68,69]. However, this is the first record of a virus belonging to the family *Paritiviridae* in *S. cerevisiae*, as well as the first report of infection by viruses of *Totiviridae* and *Mitoviridae* families in *S. bacillaris*. Despite the high infection rate among the *Saccharomyces* isolates, the number of viral species found is low, confirming the data reported in the literature for wine yeasts [29]. Particularly, the average number of viral sequences per infected isolate is low compared to what is usually reported in filamentous fungi often subjected to complex infections with viruses belonging to different families [8,11,13,23]. In this work, we identified multiple viral infections in *S. bacillaris* and in all *S. cerevisiae* strains with the exception of *S. cerevisiae* NDA21 and Lalvin RC212, infected only by a narnavirus and a totivirus, respectively. Our results highlight the presence of natural mixed infections of viruses belonging to different families in both *Saccharomyces* and non-*Saccharomyces* yeasts. Interestingly, no toti-narnavirus combinations were found in this study nor are they reported in the previous literature, although these families are the most widespread and were widely studied in both commercial and wild yeasts of oenological interest in the last fifty years. To investigate this aspect, it will be probably necessary to test a greater number of natural yeast strains not subjected to industrial selection using a high-throughput sequencing approach.

The associations of L-A totiviruses with dsRNA M satellites reported in the literature for killer strains [30,70] were confirmed in this work in all K and N commercial *S. cerevisiae* strains, while they were not found in the S strains. In particular, all the L-A2 type maintained M2 satellites while L-A-lus maintained either Mlus or M2, as reported in wild-type strains [71]. Moreover, we did not find a specific correspondence of L-BC genetic variants with L-A genotypes, confirming the data reported in the literature [30]. Considering the intra-specific nucleotide diversity for both totivirus species, the highest values calculated for L-A (22%) and for L-BC (4.5%) (Supplementary Tables S3 and S5) fall within the range reported in the literature [30]; moreover, these ScV-L-A and ScV-L-BC isolates clustered in phylogenetic groups already described (Supplementary Figures S1 and S3), confirming the distribution of different genetic types.

Toxin production was predicted in six killer (K) and six neutral (N) *Saccharomyces* commercial strains (Table 2). The variability highlighted among the M2 dsRNAs from both K and N yeasts resulted in eight amino acid substitutions distributed along the whole sequence that cannot be directly associated with possible alterations of the toxin efficacy, with the exception of the N-strain Zymaflore FX10. For this strain, a lysine-glutamic acid substitution at position 220 could reduce the killing action of the K2 toxin, as demonstrated for other amino acid substitutions in the same KR cleavage site which is determinant for the conversion of the preprotoxin into the protoxin [72]. On the other hand, M-lus from the N strain Lalvin ICV D 254 showed a defective ORF that could compromise the synthesis of the functional K-lus toxin. Being neutral yeasts classified into two main categories, inactive toxin secretors and non-secretors [35], it is possible that the neutral phenotype of the remaining N commercial strains such as Uvaferm 43 and Zymaflore VL1 involves different mechanisms as those that alter the toxin secretion.

Despite the complexity of the family *Narnaviridae* which includes also plant and animal viruses, only three narnavirus species have been described in yeast [29,73]. Their replication mechanism was thoroughly studied by reverse genetics using an infectious clone of ScNV-20S [74]. As for all narna-like viruses, the ScNV-NDA21 monopartite genome encodes only for the RdRp protein, which is closely related to the reference ScNV-20S and NV-I329 isolates (Figure 3). The intraspecific variability of *Saccharomyces* narnavirus species has not been yet fully investigated, few complete genomic sequences are available in GenBank; however, the NDA21 RdRp amino acid sequence showed a very strong correspondence with the reference isolates reported in the literature. For this reason, considering the amino acid identity value of 50% as the accepted threshold for the demarcation of species within the narnavirus genus [42], we believe that ScNV-NDA21 can be referred to as a new genetic variant of ScNV-20S.

Two undescribed mycoviruses, detected in two commercial *S. cerevisiae* strains, were closely related to members of the genus *Cryspovirus* of family *Partitiviridae*, which to date includes only distinctive protozoal dsRNA viruses isolated from *Cryptosporidium* spp. [69]. The family *Partitiviridae* currently comprises four other genera, *Alphapartitivirus*, *Betapartitivirus*, *Gammapartitivirus*, and *Deltapartitivirus* [69,75]. According to Vainio [69], genera within this family are identified based on their hosts (which is characteristic for each genus), genome and protein lengths, pairwise RdRp aa identity <24% for viruses from different genera, and separate phylogenetic groupings of RdRp aa sequences. The high RdRp and CP identity values between D254 and U43 isolates suggest the presence of a single viral species infecting *S. cerevisiae*. The high bootstrap value obtained from the RdRp phylogenetic analysis supports the inclusion of ScCV1-D254 and ScCV1-U43 in the genus *Cryspovirus* with Cryptosporidium parvum virus 1 (CSpV1) (Figure 2).

A pairwise comparison among the most closely related cryspoviruses showed that ScCV1-D254 and ScCV1-U43 had the highest identity percentages when aligned to CSpV1 (GenBank BAU19319) RdRp (37%) or to Cryptosporidium meleagridis virus CP (GenBank ABB02500) (21.8%) (Supplementary Tables S8 and S9). Both values are well below the threshold to establish a new species in the family *Partitiviridae* (aa identities of 80% for CP and 90% for RdRp) provided by ICTV guidelines [69]. Considering the distinct phylogenetic grouping of RdRp sequences, the protein lengths as well as their host specificity, we propose ScCV1-D254 and ScCV1-U43 as isolates of a new putative species belonging to the *Cryspovirus* genus, tentatively named Saccharomyces cerevisiae cryspovirus 1 (ScCV1). To our knowledge, this is the first report of a crispovirus in budding yeasts. A further analysis of the 5′-terminal and 3′-terminal genomic sequences will define their phylogenetic relationship within the family *Partitiviridae*.

Among the non-*Saccharomyces* strains, only *Starmerella bacillaris* Cz12 was infected by two putative new viral species belonging to the *Totiviridae* and the *Mitoviridae* families. *S. bacillaris* (synonym *Candida zemplinina*) is frequently isolated from grapes, musts, and wines in different parts of the world, and it is considered as one of the most promising species for enological use because of its ability to resist alcohol in advanced stages of winemaking [76]. The Cz12 strain was isolated in Sicily from grape musts [77] and selected for its ability in sequential fermentations with *S. cerevisiae* to produce wines with about 50% more glycerol than *S. cerevisiae* alone [78,79].

The first sequence identified in *S. bacillaris* showed a conserved ORF organization typical of totiviruses, including the eight conserved regions in the ORF2, and the ribosomal slippage site between the CP and RdRp ORFs [68,80]. Considering the criteria established for the demarcation of species included in the *Totiviridae* family (host specificity and amino acid identity values below 50%) [81], the virus identified here may represent a new species tentatively named SbTV1 to be included in a putative new genus within the family *Totiviridae* together with Red algae totivirus 1 (RaTV1). The second viral sequence found in *S. bacillaris* was a putative new mitovirus species showing the best correspondence with other fungal mitoviruses. The ICTV species demarcation criteria for mitoviruses indicate amino acid identity values of 40% as thresholds to discriminate among different species. In our case, identity values among SbMV1 and the tree reference species Sclerotinia sclerotiorum mitovirus 26 (SsMV26), Entomophthora muscae mitovirus 6 (EnmuMV6), and Entomophthora muscae mitovirus 29 (EnmuMV29), ranged from 28% to 32.2%, supporting the hypothesis of a new species as also shown in the Maximum likelihood phylogenetic tree (Figure 3). To our knowledge, this is the first mitovirus found in yeasts.

Our bioinformatic pipeline to identify ORFans and in specific putative viral ORFans identified three segments encoding for proteins, but one of them turned out to be a DNA encoded ORFan, therefore unlikely to be of a current virus infection; nevertheless, the origin and function of this ORFan remains an interesting future task to be completed. Even more interesting are the two segments (ORFan2 and ORFan3) that are replicated via an RNA intermediate. Given that these two segments are in a yeast isolate containing a totivirus, it is tempting to speculate that they could be accessory satellite segments. Generally, satellites conserved terminal sequences with their helper virus, and so far, our inspection did not reveal any conservation of the termini (but we have only in-silico assembly that might miss the viral 5′ and 3′ ends). In the future, we will complete RACE for the ORFan segments and possibly derive in-vitro or in-vivo transcripts to test if they are infectious in yeast.

Mycoviruses commonly cause latent infections in fungi, and in some cases, they are able to alter remarkably the hosts biology without evident symptoms, such as for the killer system in totivirus-infected strains. In 2002, Lopez [73] demonstrated that the relative abundance of the narnavirus ScNV-20S and ScNV-23S RNA copy number increased in industrial and laboratory *S. cerevisiae* strains exposed to nutritional stress conditions, without any evident correlation between the viral infection and biological characteristics of the strains. Nevertheless, beyond the amount of literature regarding these systems, the results of the virus-host interaction are still poorly understood in yeasts, particularly in the case of new viral species and yeast species other than *S. cerevisiae* with a high oenological potential. As reported in studies on unicellular organisms, viral infections can influence the virulence of their hosts [82,83]. In some cases, the virus would act as a mutual symbiont by increasing the virulence (hypervirulence), as described for viruses infecting the parasitic protozoa *Leishmania* [84], *Trichomonas* [85], and *Cryptosporidum* [86]. In other cases, a deleterious effect of the virus on the parasite could suggest a decrease in virulence (hypovirulence) such as for *Giardia* [87]. Variation in the virulence and fecundity of *Cryptosporidium parvum* isolates were reported by studies on cryptosporidiosis [86] suggesting that CsPV could have a role in the fecundity and possibly virulence of *C. parvum* by increasing the oocysts’ production.

## 5. Conclusions

This study confirmed the presence of viruses associated with useful technological characteristics of yeast strains already employed for wine production and unveiled new viral species never reported in unicellular fungi before. The presence of complex mixed infections would imply unpredictable biological effects for the host, possibly leading to the alteration of the technological characteristics of interest, particularly in the case of commercial strains. Multiple infections may induce intra-host interactions among different viruses resulting in both synergistic and antagonistic effects as described for the interactions among plant [88] and fungal [89] viruses. These aspects portend the necessity of a periodic quality control to verify the stability of viral infections within the strain of interest.

Our relatively cheap technological approach to investigating the virome associated with yeast could be employed on larger scale studies that include the natural collection of isolates from various unexplored ecological niches. Applications of high-throughput molecular techniques coupled with protocols for transferring the infection to healthy strains will unravel the effect of viral infections on yeast biology as well as on their potential technological properties. Furthermore, given the natural yeast infection by the mitovirus and a partiti-like virus, in the future we will investigate their possible ability to infect *S. cerevisiae* and therefore establish a new virus-host model system to study mitovirus and partitivirus replication.

## Figures and Tables

**Table 1:** List of yeast strains used in this study. Killer phenotype provided by the manufacturer/distributor–K, killer; S, sensitive; N, neutral; n.a., not available.

	Species	Strain	Source	Country	Killer Phenotype	Brand
1	*Saccharomyces cerevisiae*	Lalvin BA11	Grape must	Portugal	S	Lallemand
2	*Saccharomyces cerevisiae*	Lalvin ICV D254	Grape must	France	N	Lallemand
3	*Saccharomyces cerevisiae*	Lalvin Rhone L2226	Grape must	France	K	Lallemand
4	*Saccharomyces cerevisiae*	Lalvin EC1118	Grape must	France	K	Lallemand
5	*Saccharomyces cerevisiae*	Lalvin RC212	Grape must	France	S	Lallemand
6	*Saccharomyces cerevisiae*	Lalvin Rhone 2323	Grape must	France	K	Lallemand
7	*Saccharomyces cerevisiae*	Lalvin QA23	Grape must	Portugal	K	Lallemand
8	*Saccharomyces cerevisiae*	Uvaferm 43	Grape must	France	N	Lallemand
9	*Saccharomyces cerevisiae*	Zymaflore VL1	Grape must	France	N	Laffort
10	*Saccharomyces cerevisiae*	Zymaflore F15	Grape must	France	S	Laffort
11	*Saccharomyces cerevisiae*	Zymaflore X5	Breeding	n.a.	K	Laffort
12	*Saccharomyces cerevisiae*	Zymaflore FX10	Breeding	n.a.	N	Laffort
13	*Saccharomyces cerevisiae*	Fermicru VR5	Grape must	France	N	Oenobrands
14	*Saccharomyces cerevisiae*	PDM	Grape must	France	K	Maurivin
15	*Saccharomyces cerevisiae*	EZFERM44	Grape must	n.a.	N	Enartis
16	*Saccharomyces cerevisiae*	IRVO NDA21	Grape must	Italy	n.a.	Fermentis
17	*Metschnikowia pulcherrima*	IRVO Non-Sac2	Grape must	Italy	n.a.	n.a.
18	*Hanseniaspora uvarum*	IRVO Non-Sac7	Grape must	Italy	n.a.	n.a.
19	*Kluyveromyces marxianus*	IRVO Non-Sac10	Human feces	Italy	n.a.	n.a.
20	*Starmerella bacillaris* *(sin. Candida zemplinina)*	IRVO Cz12	Grape must	Italy	n.a.	n.a.
21	*Lachancea (Kluyveromyces) thermotolerans*	DBVPG 6232	Plum conserve	USSR	n.a.	n.a.
22	*Hanseniaspora guilliermondii*	DBVPG 6796	Infected nail	South Africa	n.a.	n.a.
23	*Candida stellata*	DBVPG 6714	Grapevine	Germany	n.a	n.a.
24	*Dekkera anomala*	DBVPG 4075	Beer	UK	n.a.	n.a.

**Table 2:** List of viral isolates and corresponding GenBank accession numbers. Putative new viral species are indicated by *.

Genus	Isolate	Isolate Abbreviation	GenBank Accession No.
*Totivirus*	Saccharomyces cerevisiae virus L-A-lus strain D254	ScV-LA-lus-D254	OK377006
	Saccharomyces cerevisiae virus L-A-2 strain L2226	ScV-LA2-L2226	OK377007
	Saccharomyces cerevisiae virus L-A-2 strain EC1118	ScV-LA2-EC1118	OK377008
	Saccharomyces cerevisiae virus L-A-2 strain 2323	ScV-LA2-2323	OK377009
	Saccharomyces cerevisiae virus L-A-2 strain QA23	ScV-LA2-QA23	OK377010
	Saccharomyces cerevisiae virus L-A-lus strain U43	ScV-LA-lus-U43	OK377011
	Saccharomyces cerevisiae virus L-A-2 strain VL1	ScV-LA2-VL1	OK377012
	Saccharomyces cerevisiae virus L-A-2 strain X5	ScV-LA2-X5	OK377013
	Saccharomyces cerevisiae virus L-A-2 strain FX10	ScV-LA2-FX10	OK377014
	Saccharomyces cerevisiae virus L-A-2 strain VR5	ScV-LA2-VR5	OK377015
	Saccharomyces cerevisiae virus L-A-2 strain PDM	ScV-LA2-PDM	OK377016
	Saccharomyces cerevisiae virus L-A-2 strain EZ44	ScV-LA2-EZ44	OK377017
	Saccharomyces cerevisiae virus L-BC-2 strain EZ44	ScV-LBC2-EZ44	OK377025
	Saccharomyces cerevisiae virus L-BC-2 strain PDM	ScV-LBC2-PDM	OK377024
	Saccharomyces cerevisiae virus L-BC-2 strain VR5	ScV-LBC2-VR5	OK377023
	Saccharomyces cerevisiae virus L-BC-2 strain X5	ScV-LBC2-X5	OK377022
	Saccharomyces cerevisiae virus L-BC-2 strain QA23	ScV-LBC2-QA23	OK377021
	Saccharomyces cerevisiae virus L-BC (La) strain RC212	ScV-LBC-La-RC212	OK377020
	Saccharomyces cerevisiae virus L-BC-2 strain EC1118	ScV-LBC2-EC1118	OK377019
	Saccharomyces cerevisiae virus L-BC (La) strain D254	ScV-LBC-La-D254	OK377018
	Saccharomyces cerevisiae virus satellite dsRNA Mlus strain D254	ScV-Mlus-D254	OK412909
	Saccharomyces cerevisiae virus satellite dsRNA M2 strain L2226	ScV-M2-L2226	OK412898
	Saccharomyces cerevisiae virus satellite dsRNA M2 strain EC1118	ScV-M2-EC1118	OK412899
	Saccharomyces cerevisiae virus satellite dsRNA M2 strain 2323	ScV-M2-2323	OK412900
	Saccharomyces cerevisiae virus satellite dsRNA M2 strain QA23	ScV-M2-QA23	OK412901
	Saccharomyces cerevisiae virus satellite dsRNA M2 strain U43	ScV-M2-U43	OK412902
	Saccharomyces cerevisiae virus satellite dsRNA M2 strain VL1	ScV-M2-VL1	OK412903
	Saccharomyces cerevisiae virus satellite dsRNA M2 strain X5	ScV-M2-X5	OK412904
	Saccharomyces cerevisiae virus satellite dsRNA M2 strain FX10	ScV-M2-FX10	OK412905
	Saccharomyces cerevisiae virus satellite dsRNA M2 strain VR5	ScV-M2-VR5	OK412906
	Saccharomyces cerevisiae virus satellite dsRNA M2 strain PDM	ScV-M2-PDM	OK412907
	Saccharomyces cerevisiae virus satellite dsRNA M2 strain EZ44	ScV-M2-EZ44	OK412908
	Starmerella bacillaris totivirus 1 *	SbTV1 *	OK412911
*Cryspovirus*	Saccharomyces cerevisiae cryspovirus 1 -D254 (RNA2) *	ScCV1-D254	OK412913
	Saccharomyces cerevisiae cryspovirus 1 -U43 (RNA2) *	ScCV1-U43	OK412914
	Saccharomyces cerevisiae cryspovirus 1 -D254 (RNA1) *	ScCV1-D254	OK412915
	Saccharomyces cerevisiae cryspovirus 1 -U43 (RNA1) *	ScCV1-U43	OK412916
*Narnavirus*	Saccharomyces NDA21 narnavirus	ScNV-NDA21	OK412912
*Mitovirus*	Starmerella bacillaris mitovirus 1 *	SbMV1 *	OK412910

**Table 3:** List of viral infections detected in the yeast strains. ScV-L-A, Saccharomyces cerevisiae virus L-A; ScV-M2, Saccharomyces cerevisiae virus satellite dsRNA M2; ScV-Mlus, Saccharomyces cerevisiae virus satellite dsRNA Mlus; ScV-L-BC, Saccharomyces cerevisiae virus L-BC; SbTV1, Starmerella bacillaris totivirus 1; ScCV1, Saccharomyces cerevisiae crispovirus 1; ScNV-20S, Saccharomyces cerevisiae narnavirus 20S; SbMV1, Starmerella bacillaris mitovirus 1. The corresponding threshold cycles of diagnostic real-time PCR on cDNAs are reported in brackets.

	*Totiviridae*					*Partitiviridae*	*Narnaviridae*	*Mitoviridae*
Strain	ScV-L-A	ScV-M2	ScV-Mlus	ScV-L-BC	SbTV1	ScCV1	ScNV-20S	SbMV1
**Lalvin ICV D254**	ScV-LA-lus-D254 (20.25)		ScV-Mlus-D254 (19.33)	ScV-LBC-La-D254 (26.86)		ScCV1-D254 RNA1 (19.58) RNA2, (20.13)		
**Lalvin Rhone L2226**	ScV-LA2-L2226 (23.17)	ScV-M2-L2226(21.04)						
**Lalvin EC1118**	ScV-LA2-EC118 (22.34)	ScV-M2-EC118 (20.44)		ScV-LBC2-EC1118 (26.37)				
**Lalvin RC212**				ScV-LBC-La-RC212 (26.22)				
**Lalvin** **Rhone 2323**	ScV-LA2-2323(23.05)	ScV-M2-2323 (19.05)						
**Lalvin QA23**	ScV-LA2-QA23 (22.33)	ScV-M2-QA23(18.23)		ScV-LBC2-QA23 (26.91)				
**Uvaferm 43**	ScV-LA-lus-U43 (24.28)	ScV-M2-U43 (18.18)				ScCV1-U43 (RNA1, 20.71) (RNA2, 16.30)		
**Zymaflore VL1**	ScV-LA2-VL1 (22.78)	ScV-M2-VL1 (18.61)						
**Zymaflore X5**	ScV-LA2-X5 (23.61)	ScV-M2-X5 (21.68)		ScV-LBC2-X5(27.08)				
**Zymaflore FX10**	ScV-LA2-FX10(22.51)	ScV-M2-FX10(21.33)						
**Fermicru VR5**	ScV-LA2-VR5 (22.72)	ScV-M2-VR5 (21.47)		ScV-LBC2-VR5 (27.72)				
**PDM**	ScV-LA2-PDM (23.50)	ScV-M2-PDM (21.78)		ScV-LBC2-PDM (28.30)				
**EZFERM44**	ScV-LA2-EZ44(24.38)	ScV-M2-EZ44 (20.56)		ScV-LBC2-EZ44 (28.01)				
**NDA21**							ScNV-20S- NDA21 (18.00)	
**Cz12**					SbTV1-Cz12 (20.35)			SbMV1-Cz12 (22.92)

## Data Availability

Publicly available datasets were analyzed in this study. This data can be found here: https://www.ncbi.nlm.nih.gov/popset/?term=OK377006 (accessed on 16 December 2021), https://www.ncbi.nlm.nih.gov/popset/?term=OK377025 (accessed on 16 December 2021), https://www.ncbi.nlm.nih.gov/nuccore/OK412909.1/ (accessed on 16 December 2021), https://www.ncbi.nlm.nih.gov/popset/?term=OK412898 (accessed on 16 December 2021), https://www.ncbi.nlm.nih.gov/popset/?term=OK412913 (accessed on 16 December 2021), https://www.ncbi.nlm.nih.gov/popset/?term=OK412915 (accessed on 16 December 2021), https://www.ncbi.nlm.nih.gov/nuccore/OK412912.1/ (accessed on 16 December 2021), https://www.ncbi.nlm.nih.gov/nuccore/OK412910 (accessed on 16 December 2021).

## Supplementary Materials

The following supporting information can be downloaded at: https://www.mdpi.com/article/10.3390/v14010052/s1, Figure S1: Phylogenetic analysis of ScV-LA viral isolates; Figure S2: Alignment of the K2 preprotoxins from different M dsRNAs; Figure S3: Phylogenetic analysis of ScV-L-BC viral isolates; Table S1: Specific primers designed in this study; Table S2: Sequences of isolates retrieved from the GenBank; Table S3: Nucleotide percent identity and amino acid percent identity of ScV-LA isolates for Coat protein gene; Table S4: Nucleotide percent identity and amino acid percent identity of ScV-M-2 isolates for K-2 killer toxin; Table S5: Nucleotide percent identity and amino acid percent identity of ScV-L-BC isolates for partial RdRp and CP; Table S6: Amino acid percent identities of SbTV1 for Coat Protein gene; Table S7: Amino acid percent identities of SbTV1 for RNA dependent RNA polymerase gene; Table S8: Amino acid percent identities of ScCV1 isolates for RNA dependent RNA polymerase; Table S9: Amino acid percent identities of ScCV1 isolates for Coat protein; Table S10: Amino acid identities of ScV-NV-NDA21 for RNA dependent RNA polymerase; Supplementary online Text S1: ORFan FASTA sequences.

## References

[1] Ghabrial S.A., Castón J.R., Jiang D., Nibert M.L., Suzuki N. (2015). 50-plus Years of Fungal Viruses. Virology.

[2] Myers J.M., Bonds A.E., Clemons R.A., Thapa N.A., Simmons D.R., Carter-House D., Ortanez J., Liu P., Miralles-Durán A., Desirò A. (2020). Survey of Early-Diverging Lineages of Fungi Reveals Abundant and Diverse Mycoviruses. mBio.

[3] Son M., Yu J., Kim K.-H. (2015). Five Questions about Mycoviruses What Are Mycoviruses?. PLoS Pathog..

[4] Krupovic M., Said A.G., Jiang D., Varsani A. (2016). Genomoviridae: A New Family of Widespread Single-Stranded DNA Viruses. Arch. Virol..

[5] Khalifa M.E., Macdiarmid R.M. (2021). A Mechanically Transmitted Dna Mycovirus Is Targeted by the Defence Machinery of Its Host, Botrytis Cinerea. Viruses.

[6] Ghabrial S.A., Suzuki N. (2009). Viruses of Plant Pathogenic Fungi. Annu. Rev. Phytopathol..

[7] Xie J., Jiang D. (2014). New Insights into Mycoviruses and Exploration for the Biological Control of Crop Fungal Diseases. Annu. Rev. Phytopathol..

[8] Ruiz-Padilla A., Rodríguez-Romero J., Gómez-Cid I., Pacifico D., Ayllón M.A. (2021). Novel Mycoviruses Discovered in the Mycovirome of a Necrotrophic Fungus. mBio.

[9] Sato Y., Castón J.R., Suzuki N. (2018). The Biological Attributes, Genome Architecture and Packaging of Diverse Multi-Component Fungal Viruses. Curr. Opin. Virol..

[10] Jia H., Dong K., Zhou L., Wang G., Hong N., Jiang D., Xu W. (2017). A DsRNA Virus with Filamentous Viral Particles. Nat. Commun..

[11] Mu F., Li B., Cheng S.I., Jia J., Jiang D., Fu Y., Cheng J., Lin Y., Chen T., Xie J. (2021). Nine Viruses from Eight Lineages Exhibiting New Evolutionary Modes That Co-Infect a Hypovirulent Phytopathogenic Fungus. PLoS Pathog..

[12] Chiapello M., Rodríguez-Romero J., Aylló M.A., Turina M. (2020). Analysis of the Virome Associated to Grapevine Downy Mildew Lesions Reveals New Mycovirus Lineages. Virus Evol..

[13] Picarelli M.A.S.C., Forgia M., Rivas E.B., Nerva L., Chiapello M., Turina M., Colariccio A. (2019). Extreme Diversity of Mycoviruses Present in Isolates of Rhizoctonia Solani AG2-2 LP from Zoysia Japonica from Brazil. Front. Cell. Infect. Microbiol..

[14] Crabtree A.M., Kizer E.A., Hunter S.S., van Leuven J.T., New D.D., Fagnan M.W., Rowley P.A. (2019). A Rapid Method for Sequencing Double-Stranded RNAs Purified from Yeasts and the Identification of a Potent K1 Killer Toxin Isolated from *Saccharomyces cerevisiae*. Viruses.

[15] Nerva L., Ciuffo M., Vallino M., Margaria P., Varese G.C., Gnavi G., Turina M. (2016). Multiple Approaches for the Detection and Characterization of Viral and Plasmid Symbionts from a Collection of Marine Fungi. Virus Res..

[16] Abdoulaye A.H., Foda M.F., Kotta-Loizou I. (2019). Viruses Infecting the Plant Pathogenic Fungus Rhizoctonia Solani. Viruses.

[17] Gilbert K.B., Holcomb E.E., Allscheid R.L., Carrington J.C. (2019). Hiding in Plain Sight: New Virus Genomes Discovered via a Systematic Analysis of Fungal Public Transcriptomes. PLoS ONE.

[18] Marzano S.Y.L., Domier L.L. (2016). Novel Mycoviruses Discovered from Metatranscriptomics Survey of Soybean Phyllosphere Phytobiomes. Virus Res..

[19] Donaire L., Ayllon M.A. (2017). Deep Sequencing of Mycovirus-Derived Small RNAs from Botrytis Species. Mol. Plant Pathol..

[20] Kreuze J.F., Perez A., Untiveros M., Quispe D., Fuentes S., Barker I., Simon R. (2009). Complete Viral Genome Sequence and Discovery of Novel Viruses by Deep Sequencing of Small RNAs: A Generic Method for Diagnosis, Discovery and Sequencing of Viruses. Virology.

[21] Vainio E.J., Jurvansuu J., Streng J., Rajamä M.-L., Hantula J., Valkonen J.P.T. (2015). Diagnosis and Discovery of Fungal Viruses Using Deep Sequencing of Small RNAs. J. Gen. Virol..

[22] Fischer D., Eisenberg D. (1999). Finding Families for Genomic ORFans. Bioinform. Discov. Note.

[23] Bartholomäus A., Wibberg D., Winkler A., Pühler A., Schlüter A., Varrelmann M. (2016). Deep Sequencing Analysis Reveals the Mycoviral Diversity of the Virome of an Avirulent Isolate of Rhizoctonia Solani AG-2-2 IV. PLoS ONE.

[24] Andika I.B., Wei S., Cao C., Salaipeth L., Kondo H., Sun L. (2017). Phytopathogenic Fungus Hosts a Plant Virus: A Naturally Occurring Cross-Kingdom Viral Infection. Proc. Natl. Acad. Sci. USA.

[25] Dolja V.V., Koonin E.V. (2018). Metagenomics Reshapes the Concepts of RNA Virus Evolution by Revealing Extensive Horizontal Virus Transfer. Virus Res..

[26] Nerva L., Varese G.C., Falk B.W., Turina M. (2017). Mycoviruses of an Endophytic Fungus Can Replicate in Plant Cells: Evolutionary Implications. Sci. Rep..

[27] Rowley P.A. (2017). The Frenemies within: Viruses, Retrotransposons and Plasmids That Naturally Infect Saccharomyces Yeasts. Yeast.

[28] Zhao R.Y. (2017). Yeast for Virus Research. Microb. Cell.

[29] Mardanov A.V., Beletsky A.V., Tanashchuk T.N., Kishkovskaya S.A., Ravin N.V. (2020). A Novel Narnavirus from a *Saccharomyces cerevisiae* Flor Strain. Arch. Virol..

[30] Rodríguez-Cousiño N., Esteban R. (2017). Relationships and Evolution of Double-Stranded RNA Totiviruses of Yeasts Inferred from Analysis of L-A-2 and L-BC Variants in Wine Yeast Strain Populations. Appl. Environ. Microbiol..

[31] Rodríguez-Cousiño N., Gómez P., Esteban R. (2017). Variation and Distribution of L-A Helper Totiviruses in Saccharomyces Sensu Stricto Yeasts Producing Different Killer Toxins. Toxins.

[32] Rodríguez-Cousiño N., Maqueda M., Ambrona J., Zamora E., Esteban R., Ramírez M. (2011). A New Wine *Saccharomyces cerevisiae* Killer Toxin (Klus), Encoded by a Double-Stranded RNA Virus, with Broad Antifungal Activity Is Evolutionarily Related to a Chromosomal Host Gene. Appl. Environ. Microbiol..

[33] Schmitt M.J., Breinig F. (2006). Yeast Viral Killer Toxins: Lethality and Self-Protection. Nat. Rev. Microbiol..

[34] van Vuuren H.J.J., Wingfield B.D. (1986). Killer Yeasts-Cause of Stuck Fermentations in a Wine Cellar. S. Afr. J. Enol. Vitic..

[35] Bussey H., Sacks W., Galley D., Saville D. (1982). Yeast Killer Plasmid Mutations Affecting Toxin Secretion and Activity and Toxin Immunity Function. Mol. Cell. Biol..

[36] Boynton P.J. (2019). The Ecology of Killer Yeasts: Interference Competition in Natural Habitats. Yeast.

[37] Fredericks L.R., Lee M.D., Crabtree A.M., Boyer J.M., Kizer E.A., Taggart N.T., Roslund C.R., Hunter S.S., Kennedy C.B., Willmore C.G. (2021). The Species-Specific Acquisition and Diversification of a K1-like Family of Killer Toxins in Budding Yeasts of the Saccharomycotina. PLoS Genet..

[38] Melvydas V., Bružauskaitė I., Gedminienė G., Šiekštelė R. (2016). A Novel *Saccharomyces cerevisiae* Killer Strain Secreting the X Factor Related to Killer Activity and Inhibition of *S. cerevisiae* K1, K2 and K28 Killer Toxins. Indian J. Microbiol..

[39] Csoma H., Zakany N., Capece A., Romano P., Sipiczki M. (2010). Biological Diversity of Saccharomyces Yeasts of Spontaneously Fermenting Wines in Four Wine Regions: Comparative Genotypic and Phenotypic Analysis. Int. J. Food Microbiol..

[40] Ivannikova Y.V., Naumova E.S., Naumov G.I. (2007). Viral DsRNA in the Wine Yeast *Saccharomyces Bayanus* Var. Uvarum. Res. Microbiol..

[41] Kadowaki K., Halvorson H.O. (1971). Appearance of a New Species of Ribonucleic Acid During Sporulation in *Saccharomyces cerevisiae*. J. Bacteriol..

[42] Hillman B.I., Esteban R., King A., Adams M., Carstens E., Lefkowitz E. (2011). Family Narnaviridae. Virus Taxonomy: Ninth Report of the International Committee for the Taxonomy of Viruses.

[43] Vepštaitė-Monstavičė I., Lukša J., Konovalovas A., Ežerskytė D., Stanevičienė R., Strazdaitė-žielienė Ž., Serva S., Servienė E. (2018). Saccharomyces Paradoxus K66 Killer System Evidences Expanded Assortment of Helper and Satellite Viruses. Viruses.

[44] Schmiti M.J., Neuhausen F. (1994). Killer Toxin-Secreting Double-Stranded RNA Mycoviruses in the Yeasts Hanseniaspora Uvarum and Zygosaccharomyces Bailii. J. Virol..

[45] Ramírez M., Velázquez R., Maqueda M., Martínez A. (2020). Genome Organization of a New Double-Stranded RNA LA Helper Virus From Wine Torulaspora Delbrueckii Killer Yeast as Compared With Its Saccharomyces Counterparts. Front. Microbiol..

[46] Nagy P.D., Pogany J., Lin J.Y. (2014). How Yeast Can Be Used as a Genetic Platform to Explore Virus–Host Interactions: From ‘Omics’ to Functional Studies. Trends Microbiol..

[47] Sahaya Glingston R., Yadav J., Rajpoot J., Joshi N., Nagotu S. (2021). Contribution of Yeast Models to Virus Research. Appl. Microbiol. Biotechnol..

[48] Matsumura E.E., Coletta-Filho H.D., Nourin S., Falk B.W., Nerva L., Oliveira T.S., Dorta S.O., Machado M.A. (2017). Deep Sequencing Analysis of RNAs from Citrus Plants Grown in a Citrus Sudden Death–Affected Area Reveals Diverse Known and Putative Novel Viruses. Viruses.

[49] Nerva L., Varese G.C., Turina M., Humana Press (2018). Different Approaches to Discover Mycovirus Associated to Marine Organisms. Methods in Molecular Biology.

[50] Haas B.J., Papanicolaou A., Yassour M., Grabherr M., Blood P.D., Bowden J., Couger M.B., Eccles D., Li B., Lieber M. (2013). De Novo Transcript Sequence Reconstruction from RNA-Seq Using the Trinity Platform for Reference Generation and Analysis. Nat. Protoc..

[51] Altschul S.F., Madden T.L., Schäffer A.A., Zhang J., Zhang Z., Miller W., Lipman D.J. (1997). Gapped BLAST and PSI-BLAST: A New Generation of Protein Database Search Programs.

[52] Langmead B., Wilks C., Antonescu V., Charles R. (2019). Scaling Read Aligners to Hundreds of Threads on General-Purpose Processors. Bioinformatics.

[53] Li H., Durbin R. (2009). Fast and Accurate Short Read Alignment with Burrows-Wheeler Transform. Bioinformatics.

[54] Thorvaldsdóttir H., Robinson J.T., Mesirov J.P. (2013). Integrative Genomics Viewer (IGV): High-Performance Genomics Data Visualization and Exploration. Brief. Bioinform..

[55] Kumar S., Stecher G., Li M., Knyaz C., Tamura K. (2018). MEGA X: Molecular Evolutionary Genetics Analysis across Computing Platforms. Mol. Biol. Evol..

[56] Capella-Gutiérrez S., Silla-Martínez J.M., Gabaldón T. (2009). TrimAl: A Tool for Automated Alignment Trimming in Large-Scale Phylogenetic Analyses. Bioinformatics.

[57] Mizutani Y., Abraham A., Uesaka K., Kondo H., Suga H., Suzuki N., Chiba S. (2018). Novel Mitoviruses and a Unique Tymo-like Virus in Hypovirulent and Virulent Strains of the Fusarium Head Blight Fungus, Fusarium Boothii. Viruses.

[58] Jolly N.P., Varela C., Pretorius I.S. (2014). Not Your Ordinary Yeast: Non-Saccharomyces Yeasts in Wine Production Uncovered. FEMS Yeast Res..

[59] Steensels J., Meersman E., Snoek T., Saels V., Verstrepen K.J. (2014). Large-Scale Selection and Breeding to Generate Industrial Yeasts with Superior Aroma Production. Appl. Environ. Microbiol..

[60] Comitini F., Agarbati A., Canonico L., Ciani M. (2021). Yeast Interactions and Molecular Mechanisms in Wine Fermentation: A Comprehensive Review. Int. J. Mol. Sci..

[61] Padilla B., Gil J.V., Manzanares P. (2016). Past and Future of Non-Saccharomyces Yeasts: From Spoilage Microorganisms to Biotechnological Tools for Improving Wine Aroma Complexity. Front. Microbiol..

[62] Díaz M.A., Pereyra M.M., Picón-Montenegro E., Meinhardt F., Dib J.R. (2020). Killer Yeasts for the Biological Control of Postharvest Fungal Crop Diseases. Microorganisms.

[63] Muccilli S., Restuccia C. (2015). Bioprotective Role of Yeasts. Microorganisms.

[64] Drinnenberg I.A., Fink G.R., Bartel D.P. (2011). Compatibility with Killer Explains the Rise of RNAi-Deficient Fungi. Science.

[65] Chang S.L., Leu J.Y., Chang T.H. (2015). A Population Study of Killer Viruses Reveals Different Evolutionary Histories of Two Closely Related Saccharomyces Sensu Stricto Yeasts. Mol. Ecol..

[66] Ramírez M., Velázquez R., López-Piñeiro A., Naranjo B., Roig F., Llorens C. (2017). New Insights into the Genome Organization of Yeast Killer Viruses Based on “Atypical” Killer Strains Characterized by High-Throughput Sequencing. Toxins.

[67] Mackay I.M., Arden K.E., Nitsche A. (2002). survey and summary Real-Time PCR in Virology. Nucleic Acids Res..

[68] Chiba Y., Tomaru Y., Shimabukuro H., Kimura K., Hirai M., Takaki Y., Hagiwara D., Nunoura T., Urayama S.I. (2020). Viral Rna Genomes Identified from Marine Macroalgae and a Diatom. Microbes Environ..

[69] Vainio E.J., Chiba S., Ghabrial S.A., Maiss E., Roossinck M., Sabanadzovic S., Suzuki N., Xie J., Nibert M. (2018). ICTV Virus Taxonomy Profile: Partitiviridae. J. Gen. Virol..

[70] Aitmanaitė L., Konovalovas A., Medvedevas P., Servienė E., Serva S. (2021). Specificity Determination in *Saccharomyces Cerevisiae* Killer Virus Systems. Microorganisms.

[71] Lukša J., Ravoitytė B., Aitmanaitė A., Lina K., Butenko A., Yurchenko V., Serva S., Servienė E. (2017). Different Metabolic Pathways Are Involved in Response of *Saccharomyces cerevisiae* to L-A and M Viruses. Toxins.

[72] Dignard D., Whiteway M., Germain D., Tessier D., Thomas D.Y. (1991). Expression in Yeast of a EDNA Copy of the K2 Killer Toxin Gene. Mol. Genet. Genom..

[73] Lopez V., Gil R., Carbonell J.V., Navarro A. (2002). Occurrence of 20S RNA and 23S RNA Replicons in Industrial Yeast Strains and Their Variation under Nutritional Stress Conditions. Yeast.

[74] Esteban R., Vega L., Fujimura T. (2005). Launching of the Yeast 20 S RNA Narnavirus by Expressing the Genomic or Antigenomic Viral RNA in Vivo. J. Biol. Chem..

[75] Nibert M.L., Ghabrial S.A., Maiss E., Lesker T., Vainio E.J., Jiang D., Suzuki N. (2014). Taxonomic Reorganization of Family Partitiviridae and Other Recent Progress in Partitivirus Research. Virus Res..

[76] Englezos V., Giacosa S., Rantsiou K., Rolle L., Cocolin L. (2017). Starmerella Bacillaris in Winemaking: Opportunities and Risks. Curr. Opin. Food Sci..

[77] Romancino D.P., di Maio S., Muriella R., Oliva D. (2008). Analysis of Non-Saccharomyces Yeast Populations Isolated from Grape Musts from Sicily (Italy). J. Appl. Microbiol..

[78] di Maio S., Genna G., Gandolfo V., Amore G., Ciaccio M., Oliva D. (2012). Presence of Candida Zemplinina in Sicilian Musts and Selection of a Strain for Wine Mixed Fermentations. S. Afr. J. Enol. Vitic..

[79] Giaramida P., Ponticello G., di Maio1 S., Squadrito M., Genna G., Barone E., Scacco A., Corona O., Amore G., di Stefano R. (2013). Candida Zemplinina for Production of Wines with Less Alcohol and More Glycerol. S. Afr. J. Enol. Vitic..

[80] Jamal A., Sato Y., Shahi S., Shamsi W., Kondo H., Suzuki N. (2019). Novel Victorivirus from a Pakistani Isolate of Alternaria Alternata Lacking a Typical Translational Stop/Restart Sequence Signature. Viruses.

[81] Wickner R.B., Ghabrial S.A., Nibert M.L., Patterson J.L., Wang C.C., King A.M.Q., Adams M.J., Carstens E.B., Lefkowitz E.J. (2012). Totiviridae. Virus Taxonomy: Classification and Nomenclature of Viruses: Ninth Report of the International Committee on Taxonomy of Viruses.

[82] Gómez-Arreaza A., Haenni A.L., Dunia I., Avilán L. (2017). Viruses of Parasites as Actors in the Parasite-Host Relationship: A “Ménage à Trois. ” Acta Trop..

[83] Roossinck M.J. (2011). The Good Viruses: Viral Mutualistic Symbioses. Nat. Rev. Microbiol..

[84] Ives A., Ronet C., Prevel F., Ruzzante G., Fuertes-Marraco S., Schutz F., Zangger H., Revaz-Breton M., Lye L.F., Hickerson S.M. (2011). Leishmania RNA Virus Controls the Severity of Mucocutaneous Leishmaniasis. Science.

[85] Fichorova R.N., Lee Y., Yamamoto H.S., Takagi Y., Hayes G.R., Goodman R.P., Chepa-Lotrea X., Buck O.R., Murray R., Kula T. (2012). Endobiont Viruses Sensed by the Human Host-Beyond Conventional Antiparasitic Therapy. PLoS ONE.

[86] Jenkins M.C., Higgins J., Abrahante J.E., Kniel K.E., O’Brien C., Trout J., Lancto C.A., Abrahamsen M.S., Fayer R. (2008). Fecundity of Cryptosporidium Parvum Is Correlated with Intracellular Levels of the Viral Symbiont CPV. Int. J. Parasitol..

[87] Miller R.L., Wang A.L., Wang C.C. (1988). Purification and Characterization of the Giardia Lamblia Double-Stranded RNA Virus. Mol. Biochem. Parasitol..

[88] Syller J. (2012). Facilitative and Antagonistic Interactions between Plant Viruses in Mixed Infections. Mol. Plant Pathol..

[89] Dobbs E., Deakin G., Bennett J., Fleming-Archibald C., Jones I., Grogan H., Burton K., Dobbs C.E. (2021). Viral Interactions and Pathogenesis during Multiple Viral Infections in Agaricus Bisporus. mBio.

