# Supplementary material for: Identification and Molecular Characterization of Novel Mycoviruses in Saccharomyces and Non-Saccharomyces Yeasts of Oenological Interest
Source: Viruses. 2021 Dec 29;14(1):52. doi: 10.3390/v14010052 (PMC8778689; doi:10.3390/v14010052)
Supplement: Supplementary file 1 [file viruses-14-00052-s001.zip › viruses-1472465-supplementary/Supplementary Figure S2.pdf]

Supplementary Figure S2: Alignment of the K2 preprotoxins from different M dsRNAs. Amino acid substitutions are highlighted.

```
ScV_M2_L2226      1 ------SLMQDELTLGEPATQARMCVRLLRFFIGLTITAFIIAACIIKSATGGSGYSNAV
ScV_M2_EC1118     1 ------SLVQDELTLGEPATRARMCVRLLRFFIGLTITAFIIAACIIKSATGGSGYSNAV
ScV_M2_2323       1 ------SLMQDELTLGEPATQARMCVRLLRFFIGLTITAFIIAACIIKSATGGSGYSNAV
ScV_M2_QA23       1 ------SLVQDELTLGEPATRARMCVRLLRFFIGLTITAFIIAACIIKSATGGSGYSNAV
ScV_M2_U43        1 ------SLMQDELTLGEPATQARMCVRLLRFFIGLTITAFIIAACIIKSATGGSGYSNAV
ScV_M2_VL1        1 ------SLVQDELTLGEPATRARMCVRLLRFFIGLTITAFIIAACIIKSATGGSGYSNAV
ScV_M2_X5         1 ------SLVQDELTLGEPATRARMCVRLLRFFIGLTITAFIIAACIIKSATGGSGYSNAV
ScV_M2_FX10       1 ------SLVQDELTLGEPATRARMCVRLLRFFIGLTITAFIIAACIIKSATGGSGYSNAV
ScV_M2_VR5        1 ------SLVQDELTLGEPATRARMCVRLLRFFIGLTITAFIIAACIIKSATGGSGYSNAV
ScV_M2_PDM        1 ------SLVQDELTLGEPATRARMCVRLLRFFIGLTITAFIIAACIIKSATGGSGYSNAV
ScV_M2_EZ44       1 ------SLVQDELTLGEPATRARMCVRLLRFFIGLTITAFIIAACIIKSATGGSGYSNAV
Scv-M2(X54154)    1 ------SLVQDELTLGEPATRARMCVRLLRFFIGLTITAFIIAACIIKSATGGSGYSNAV
Scv-M2(X56604)    1 ------SLMQDELTLGEPATQARMCVRLLRFFIGLTITAFIIAACIIKSATGGSGYSKAV
Scv-M2-4          1 ------SLMQDELTLGEPATQARMCVRLLRFFIGLTITAFVIAACIIKSATGGSGYSNAV
K2toxin(CAA38093) 1 MKETTTSLVQDELTLGEPATRARMCVRLLRFFIGLTITAFIIAACIIKSATGGSGYSNAV
K2toxin(CAA39941) 1 MKETTTSLMQDELTLGEPATQARMCVRLLRFFIGLTITAFIIAACIIKSATGGSGYSKAV

ScV_M2_L2226      55 AVRGEADTPSTIVGQLVERGGFQAWAVGAGIYLFAKIAYDTSKVTAAVCNPEALIAITSY
ScV_M2_EC1118     55 AVRGEVDTPSTIVGQLVERGGFQAWAVGAGIYLFAKIAYDTSKVTAAVCNPEALIAITSY
ScV_M2_2323       55 AVRGEADTPSTIVGQLVERGGFQAWAVGAGIYLFAKIAYDTSKVTAAVCNPEALIAITSY
ScV_M2_QA23       55 AVRGEVDTPSTIVGQLVERGGFQAWAVGAGIYLFAKIAYDTSKVTAAVCNPEALIAITSY
ScV_M2_U43        55 AVRGEADTPSTIVGQLVERGGSQAWAAGAGIYLFAKIAYDTSKVTAAVCNPEALIAITSY
ScV_M2_VL1        55 AVRGEADTPSTIVGQLVERGGFQAWAVGAGIYLFAKIAYDTSKVTAAVCNPEALIAITSY
ScV_M2_X5         55 AVRGEVDTPSTIVGQLVERGGFQAWAVGAGIYLFAKIAYDTSKVTAAVCNPEALIAITSY
ScV_M2_FX10       55 AVRGEADTPSTIVGQLVERGGFQAWAVGAGIYLFAKIAYDTSKVTAAVCNPEALIAITSY
ScV_M2_VR5        55 AVRGEVDTPSTIVGQLVERGGFQAWAVGAGIYLFAKIAYDTSKVTAAVCNPEALIAITSY
ScV_M2_PDM        55 AVRGEVDTPSTIVGQLVERGGFQAWAVGAGIYLFAKIAYDTSKVTAAVCNPEALIAITSY
ScV_M2_EZ44       55 AVRGEVDTPSTIVGQLVERGGFQAWAVGAGIYLFAKIAYDTSKVTAAVCNPEALIAITSY
Scv-M2(X54154)    55 AVRGEADTPSTIVGQLVERGGFQAWAVGAGIYLFAKIAYDTSKVTAAVCNPEALIAITSY
Scv-M2(X56604)    55 AVRGEADTPSTIVGQLVERGGFQAWAVGAGIYLFAKIAYDTSKVTAAVCNPEALIAITSY
Scv-M2-4          55 AVWGEADTPSTIVGQLVERGGFQAWAVGAGIYLFAKIAYDTSKVTAAVCNPEALIAITSY
K2toxin(CAA38093) 61 AVRGEADTPSTIVGQLVERGGFQAWAVGAGIYLFAKIAYDTSKVTAAVCNPEALIAITSY
K2toxin(CAA39941) 61 AVRGEADTPSTIVGQLVERGGFQAWAVGAGIYLFAKIAYDTSKVTAAVCNPEALIAITSY

ScV_M2_L2226      115 VAYAPTLCAGAYVIGAMSGAMSAGLALYAGYKGWQWDGPGGMAEREDVASFYSPLLNNTL
ScV_M2_EC1118     115 VAYAPTLCAGAYVIGAMSGAMSAGLALYAGYKGWQWGGPGGMAEREDVASFYSPLLNNTL
ScV_M2_2323       115 VAYAPTLCAGAYVIGAMSGAMSAGLALYAGYKGWQWDGPGGMAEREDVASFYSPLLNNTL
ScV_M2_QA23       115 VAYAPTLCAGAYVIGAMSGAMSAGLALYAGYKGWQWGGPGGMAEREDVASFYSPLLNNTL
ScV_M2_U43        115 VAYAPTLCAGAYVIGAMSGAMSAGLALYAGYKGWQWDGPGGMAEREDVASFYSPLLNNTL
ScV_M2_VL1        115 VAYAPTLCAGAYVIGAMSGAMSAGLALYAGYKGWQWGGPEGMAEREDVASFYSPLLNNTL
ScV_M2_X5         115 VAYAPTLCAGAYVIGAMSGAMSAGLALYAGYKGWQWGGPGGMAEREDVASFYSPLLNNTL
ScV_M2_FX10       115 AAYAPTLCAGAYVIGAMSGAMSAGLALYAGYKGWQWGGPGGMAEREDVASFYSPLLNNTL
ScV_M2_VR5        115 VAYAPTLCAGAYVIGAMSGAMSAGLALYAGYKGWQWGGPGGMAEREDVASFYSPLLNNTL
ScV_M2_PDM        115 VAYAPTLCAGAYVIGAMSGAMSAGLALYAGYKGWQWGGPGGMAEREDVASFYSPLLNNTL
ScV_M2_EZ44       115 VAYAPTLCAGAYVIGAMSGAMSAGLALYAGYKGWQWGGPGGMAEREDVASFYSPLLNNTL
Scv-M2(X54154)    115 VAYAPTLCAGAYVIGAMSGAMSAGLALYAGYKGWQWSGPGGMAEREDVASFYSPLLNNTL
Scv-M2(X56604)    115 VAYAPTLCAGAYVIGAMSGAMSAGLALYAGYKGWQWGGPGGMAEREDVASFYSPLLNNTL
Scv-M2-4          115 VAYAPTLCAGAYVIGAMSGAMSAGLALYAGYKGWQWGGPGGMAEREDVASFYSPLLNNTL
K2toxin(CAA38093) 121 VAYAPTLCAGAYVIGAMSGAMSAGLALYAGYKGWQWSGPGGMAEREDVASFYSPLLNNTL
K2toxin(CAA39941) 121 VAYAPTLCAGAYVIGAMSGAMSAGLALYAGYKGWQWGGPGGMAEREDVASFYSPLLNNTL

ScV_M2_L2226      175 YVGGDHTADYDSELATILGSVYNDVVHLGVYYDNSTGIVKRDSRPSMISWTVLHDNMMIT
ScV_M2_EC1118     175 YVGGDHTADYDSELATILGSVYNDVVHLGVYYDNSTGIVKRDSRPSMISWTVLHDNMMIT
ScV_M2_2323       175 YVGGDHTADYDSELATILGSVYNDVVHLGVYYDNSTGIVKRDSRPSMISWTVLHDNMMIT
ScV_M2_QA23       175 YVGGDHTADYDSELATILGSVYNDVVHLGVYYDNSTGIVKRDSRPSMISWTVLHDNMMIT
ScV_M2_U43        175 YVGGDHTADYDSELATILGSVYDDVVHLGVYYDNSTGIVKRDSRPSMISWTVLHDNMMIT
ScV_M2_VL1        175 YVGGDHTADYDSELATILGSVYNDVVHLGVYYDNSTGIVKRDSRPSMISWTVLHDNMMIT
ScV_M2_X5         175 YVGGDHTADYDSELATILGSVYNDVVHLGVYYDNSTGIVKRDSRPSMISWTVLHDNMMIT
ScV_M2_FX10       175 YVGGDHTADYDSELATILGSVYNDVVHLGVYYDNSTGIVERDSRPSMISWTVLHDNMMIT
ScV_M2_VR5        175 YVGGDHTADYDSELATILGSVYNDVVHLGVYYDNSTGIVKRDSRPSMISWTVLHDNMMIT
ScV_M2_PDM        175 YVGGDHTADYDSELATILGSVYNDVVHLGVYYDNSTGIVKRDSRPSMISWTVLHDNMMIT
ScV_M2_EZ44       175 YVGGDHTADYDSELATILGSVYNDVVHLGVYYDNSTGIVKRDSRPSMISWTVLHDNMMIT
Scv-M2(X54154)    175 YVGGDHTADYDSELATILGSVYNDVVHLGVYYDNSTGIVKRDSRPSMTSWTVLHDNMMIT
Scv-M2(X56604)    175 YVGGDHTADYDSELATILGSVYNDVVHLGVYYDNSTGIVKRDSRPSMISWTVLHDNMMIT
Scv-M2-4          175 YVGGDHTADYDSELATILGSVYNDVVHLGVYYDNSTGIVKRDSRPSMISWTVLHDNMMIT
K2toxin(CAA38093) 181 YVGGDHTADYDSELATILGSVYNDVVHLGVYYDNSTGIVKRDSRPSMTSWTVLHDNMMIT
```

```
K2toxin(CAA39941)181 YVGGDHTADYDSELATILGSVYNDVVHLGVYYDNSTGIVKRDSRPSMISWTVLHDNMMIT

ScV_M2_L2226      235 SYHRPDQLSAAATAYKAYATNTTRVGKRQDGEWVSYSVYGENVDYERYPVAHLQEEADAC
ScV_M2_EC1118     235 SYHRPDQLGAAATAYKAYATNTTRVGKRQDGEWVSYSVYGENVDYERYPVAHLQEEADAC
ScV_M2_2323       235 SYHRPDQLSAAATAYKAYATNTTRVGKRQDGEWVSYSVYGENVDYERYPVAHLQEEADAC
ScV_M2_QA23       235 SYHRPDQLGAAATAYKAYATNTTRVGKRQDGEWVSYSVYGENVDYERYPVAHLQEEADAC
ScV_M2_U43        235 SYHRPDQLSAAATAYKAYATNTTRVGKRQDGEWVSYSVYGENVDYERYPVAHLQEEADAC
ScV_M2_VL1        235 SYHRPDQLGAAATAYKAYATNTTRVGKRQDGEWVSYSVYGENVDYERYPVAHLQEEADAC
ScV_M2_X5         235 SYHRPDQLGAAATAYKAYATNTTRVGKRQDGEWVSYSVYGENVDYERYPVAHLQEEADAC
ScV_M2_FX10       235 SYHRPDQLGAAATAYKAYATNTTRVGKRQDGEWVSYSVYGENVDYERYPVAHLQEEADAC
ScV_M2_VR5        235 SYHRPDQLGAAATAYKAYATNTTRVGKRQDGEWVSYSVYGENVDYERYPVAHLQEEADAC
ScV_M2_PDM        235 SYHRPDQLGAAATAYKAYATNTTRVGKRQDGEWVSYSVYGENVDYERYPVAHLQEEADAC
ScV_M2_EZ44       235 SYHRPDQLGAAATAYKAYATNTTRVGKRQDGEWVSYSVYGENVDYERYPVAHLQEEADAC
Scv-M2(X54154)    235 SYHRPDQLGAAATAYKAYATNTTRVGKRQDGEWVSYSVYGENVDYERYPVAHLQEEADAC
Scv-M2(X56604)    235 SYHRPDQLGAAATAYKAYTTNTTRVGKRQDGEWVSYSVYGENVDYERYPVAHLQEEADAC
Scv-M2-4          235 SYHRPDQLGAAATAYKAYATNTTRVGKRQDGEWVSYSVYGENVDYERYPVAHLQEEADAC
K2toxin(CAA38093)241 SYHRPDQLGAAATAYKAYATNTTRVGKRQDGEWVSYSVYGENVDYERYPVAHLQEEADAC
K2toxin(CAA39941)241 SYHRPDQLGAAATAYKAYTTNTTRVGKRQDGEWVSYSVYGENVDYERYPVAHLQEEADAC

ScV_M2_L2226      295 YESLGNMITSQVQPCTQRECYAMDQKVCAAVGFSSDAGVNSAMVGEAYFYAYGGVDGECD
ScV_M2_EC1118     295 YESLGNMITSQVQPCTQRECYAMDQKVCAAVGFSSDAGVNSAMVGEAYFYAYGGVDGECD
ScV_M2_2323       295 YESLGNMITSQVQPCTQRGCYAMDQKVCAAVGFSSDAGVNSAMVGEAYFYAYGGVDGECD
ScV_M2_QA23       295 YESLGNMITSQVQPCTQRECYAMDQKVCAAVGFSSDAGVNSAMVGEAYFYAYGGVDGECD
ScV_M2_U43        295 YESLGNMITSQVQPCTQRECYAMDQKVCAAVGFSSDAGVNSAMVGEAYFYAYGGVDGECD
ScV_M2_VL1        295 YESLGNMITSQVQPCTQRECYAMDQKVCAAVGFSSDAGVNSAMVGEAYFYAYGGVDGECD
ScV_M2_X5         295 YESLGNMITSQVQPCTQRECYAMDQKVCAAVGFSSDAGVNSAMVGEAYFYAYGGVDGECD
ScV_M2_FX10       295 YESLGNMITSQVQPCTQRECYAMDQKVCAAVGFSSDAGVNSAMVGEAYFYAYGGVDGECD
ScV_M2_VR5        295 YESLGNMITSQVQPCTQRECYAMDQKVCAAVGFSSDAGVNSAMVGEAYFYAYGGVDGECD
ScV_M2_PDM        295 YESLGNMITSQVQPCTQRECYAMDQKVCAAVGFSSDAGVNSAMVGEAYFYAYGGVDGECD
ScV_M2_EZ44       295 YESLGNMITSQVQPCTQRECYAMDQKVCAAVGFSSDAGVNSAMVGEAYFYAYGGVDGECD
Scv-M2 (X54154)   295 YESLGNMITSQVQPCTQRECYAMDQKVCAAVGFSSDAGVNSAMVGEAYFYAYGGVDGECD
Scv-M2 (X56604)   295 YESLGNMITSQVQPCTQRECYAMDQKVCAAVGFSSDAGVNSAMVGEAYFYAYGGVDGECD
Scv-M2-4          295 YESLGNMITSQVQPGTQRECYAMDQKVCAAVGFSSDAGVNSAIVGEAYFYAYGGVDGECD
K2toxin(CAA38093)301 YESLGNMITSQVQPCTQRECYAMDQKVCAAVGFSSDAGVNSAMVGEAYFYAYGGVDGECD
K2toxin(CAA39941)301 YESLGNMITSQVQPCTQRECYAMDQKVCAAVGFSSDAGVNSAMVGEAYFYAYGGVDGECD

ScV_M2_L2226      355 S-
ScV_M2_EC1118     355 S-
ScV_M2_2323       355 S-
ScV_M2_QA23       355 S-
ScV_M2_U43        355 S-
ScV_M2_VL1        355 S-
ScV_M2_X5         355 S-
ScV_M2_FX10       355 S-
ScV_M2_VR5        355 S-
ScV_M2_PDM        355 S-
ScV_M2_EZ44       355 S-
Scv-M2 (X54154)   355 S-
Scv-M2 (X56604)   355 S-
Scv-M2-4          355 S-
K2toxin(CAA38093)361 SG
K2toxin(CAA39941)361 SG
```
