# Supplementary material for: Identification and Molecular Characterization of Novel Mycoviruses in Saccharomyces and Non-Saccharomyces Yeasts of Oenological Interest
Source: Viruses. 2021 Dec 29;14(1):52. doi: 10.3390/v14010052 (PMC8778689; doi:10.3390/v14010052)

Supplementary Figure S3: Phylogenetic analysis of ScV-L-BC viral isolates. Maximum-likelihood tree was constructed based on the alignment of a 801 bp-long fragment covering partial RdRp and CP genes. Bootstrap values reported at the nodes are from 1000 replicates. Sequences determined in this work are indicated by ♦.

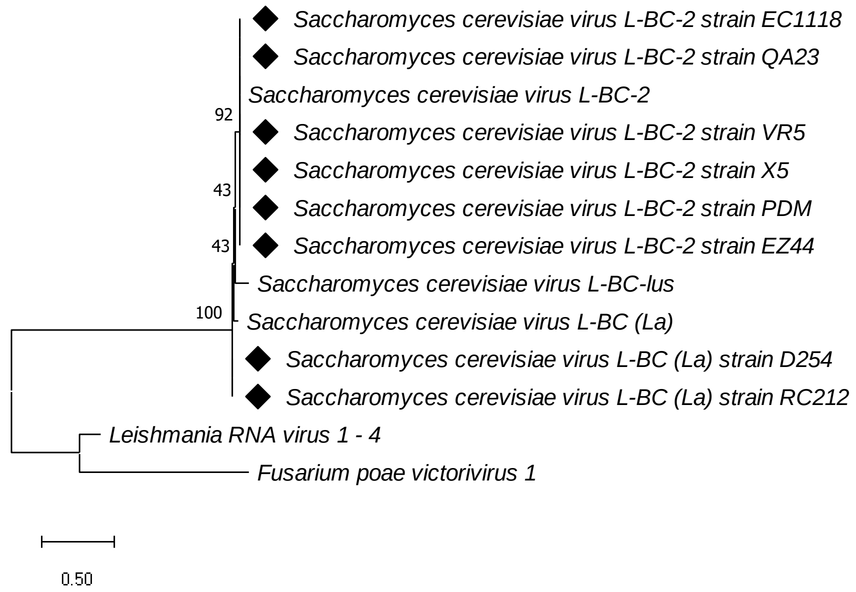

Supplement: Supplementary file 1 [file viruses-14-00052-s001.zip › viruses-1472465-supplementary/Supplementary Figure S3.pdf]
