# Supplementary material for: Identification and Molecular Characterization of Novel Mycoviruses in Saccharomyces and Non-Saccharomyces Yeasts of Oenological Interest
Source: Viruses. 2021 Dec 29;14(1):52. doi: 10.3390/v14010052 (PMC8778689; doi:10.3390/v14010052)
Supplement: Supplementary file 1 [file viruses-14-00052-s001.zip › viruses-1472465-supplementary/Supplementary Table S10.pdf]

Supplementary Table S10: Amino acid identities of ScV-NV-NDA21 for RNA dependent RNA polymerase.

	ScNV-23S (NP_660177)	ScNV-I329 (QFU28543)	ScV-NV-NDA21 (OK412912)	ScNV-20S (AAA47824)	ScNV-20S (NP_660178)
ScNV-23S (NP_660177)	100%				
ScNV-I329 (QFU28543)	29,5%	100%			
ScV-NV-NDA21 (OK412912)	30,0%	55,9%	100%		
ScNV-20S (AAA47824)	30,2%	55,8%	98,6%	100%	
ScNV-20S (NP_660178)	30,2%	56,2%	98,7%	99,9%	100%
