# Supplementary material for: Identification and Molecular Characterization of Novel Mycoviruses in Saccharomyces and Non-Saccharomyces Yeasts of Oenological Interest
Source: Viruses. 2021 Dec 29;14(1):52. doi: 10.3390/v14010052 (PMC8778689; doi:10.3390/v14010052)
Supplement: Supplementary file 1 [file viruses-14-00052-s001.zip › viruses-1472465-supplementary/Supplementary Table S3.pdf]

Supplementary Table S3: Nucleotide percent identity (below diagonal) and amino acid percent identity (above diagonal) of ScV-LA isolates for Coat protein gene.

	ScV-LA-lus-D254 (OK377006)	ScV-LA2-L2226 (OK377007)	ScV-LA2-EC1118 (OK377008)	ScV-LA2-2323 (OK377009)	ScV-LA2-QA23 (OK377010)	ScV-LA-lus-U43 (OK377011)	ScV-LA2-VL1 (OK377012)	ScV-LA2-X5 (OK377013)	ScV-LA2-FX10 (OK377014)	ScV-LA2-VR5 (OK377015)	ScV-LA2-PDM (OK377016)	ScV-LA2-EZ44 (OK377017)	ScV-LA2_8F-13 (KC677754)	ScV-LA-lus (JN819511)	ScV-LA1-1 (KX601068)	ScV-LA28 (KU845301)
ScV-LA-lus-D254 (OK377006)	100%	96,3%	95,8%	96,3%	95,8%	97,2%	96,3%	95,8%	96,3%	95,8%	95,8%	95,8%	96,3%	99,5%	93,0%	90,2%
ScV-LA2-L2226 (OK377007)	79,3%	100%	99,5%	100,0%	99,5%	95,8%	100,0%	99,5%	100,0%	99,5%	99,5%	99,5%	100,0%	95,8%	93,5%	92,1%
ScV-LA2-EC1118 (OK377008)	79,3%	99,7%	100%	99,5%	100,0%	95,3%	99,5%	100,0%	99,5%	100,0%	100,0%	100,0%	99,5%	95,3%	93,0%	91,6%
ScV-LA2-2323 (OK377009)	79,3%	100,0%	99,7%	100%	99,5%	95,8%	100,0%	99,5%	100,0%	99,5%	99,5%	99,5%	100,0%	95,8%	93,5%	92,1%
ScV-LA2-QA23 (OK377010)	79,1%	99,5%	99,8%	99,5%	100%	95,3%	99,5%	100,0%	99,5%	100,0%	100,0%	100,0%	99,5%	95,3%	93,0%	91,6%
ScV-LA-lus-U43 (OK377011)	85,3%	77,6%	77,6%	77,6%	77,7%	100%	95,8%	95,3%	95,8%	95,3%	95,3%	95,3%	95,8%	96,7%	93,0%	90,7%
ScV-LA2-VL1 (OK377012)	79,1%	99,5%	99,2%	99,5%	99,1%	77,7%	100%	99,5%	100,0%	99,5%	99,5%	99,5%	100,0%	95,8%	93,5%	92,1%
ScV-LA2-X5 (OK377013)	79,3%	99,7%	100,0%	99,7%	99,8%	77,6%	99,2%	100%	99,5%	100,0%	100,0%	100,0%	99,5%	95,3%	93,0%	91,6%
ScV-LA2-FX10 (OK377014)	79,4%	99,8%	99,5%	99,8%	99,4%	77,7%	99,4%	99,5%	100%	99,5%	99,5%	99,5%	100,0%	95,8%	93,5%	92,1%
ScV-LA2-VR5 (OK377015)	79,3%	99,7%	100,0%	99,7%	99,8%	77,6%	99,2%	100,0%	99,5%	100%	100,0%	100,0%	99,5%	95,3%	93,0%	91,6%
ScV-LA2-PDM (OK377016)	79,3%	99,7%	100,0%	99,7%	99,8%	77,6%	99,2%	100,0%	99,5%	100%	100%	100%	99,5%	95,3%	93,0%	91,6%
ScV-LA2-EZ44 (OK377017)	79,3%	99,7%	100,0%	99,7%	99,8%	77,6%	99,2%	100,0%	99,5%	100%	100%	100%	99,5%	95,3%	93,0%	91,6%
ScV-LA2_8F-13 (KC677754)	79,4%	99,5%	99,2%	99,5%	99,1%	77,4%	99,1%	99,2%	99,4%	99,2%	99,2%	99,2%	100%	95,8%	93,5%	92,1%
ScV-LA-lus (JN819511)	98,6%	79,0%	79,0%	79,0%	78,8%	84,9%	78,8%	79,0%	79,1%	79,0%	79,0%	79,0%	79,1%	100%	92,6%	89,8%
ScV-LA1-1 (KX601068)	76,7%	76,8%	76,8%	76,8%	77,0%	76,8%	77,0%	76,8%	76,7%	76,8%	76,8%	76,8%	76,7%	76,5%	100%	94,4%
ScV-LA28 (KU845301)	79,0%	78,2%	77,9%	78,2%	77,7%	79,4%	77,9%	77,9%	78,2%	77,9%	77,9%	77,9%	77,7%	78,7%	79,0%	100%
