# Supplementary material for: Identification and Molecular Characterization of Novel Mycoviruses in Saccharomyces and Non-Saccharomyces Yeasts of Oenological Interest
Source: Viruses. 2021 Dec 29;14(1):52. doi: 10.3390/v14010052 (PMC8778689; doi:10.3390/v14010052)
Supplement: Supplementary file 1 [file viruses-14-00052-s001.zip › viruses-1472465-supplementary/Supplementary Table S4.pdf]

Supplementary Table S4: Nucleotide percent identity (below diagonal) and amino acid percent identity (above diagonal) of ScV-M-2 isolates for K-2 killer toxin.

	ScV-M2-L2226 (OK412898)	ScV-M2-EC1118 (OK412899)	ScV-M2-2323 (OK412900)	ScV-M2- QA23 (OK412901)	ScV-M2-U43 (OK412902)	ScV-M2- VL1(OK412903)	ScV-M2- X5 (OK412904)	ScV-M2- FX10 (OK412905)	ScV-M2-VR5 (OK412906)	ScV-M2- PDM (OK412907)	ScV-M2- EZ44 (OK412908)	K2 type killer virus (X54154)	mRNA for K2 killer toxin (X56604)	*S.cerevisiae* K-2 gene (X56987)	ScV-M2-4 (MF957266)
ScV-M2-L2226 (OK412898)	100%	98,6%	99,7%	98,6%	99,2%	98,6%	98,6%	98,3%	98,6%	98,6%	98,6%	98,6%	98,9%	72,6%	98,3%
ScV-M2-EC1118 (OK412899)	99,1%	100%	98,3%	100,0%	97,7%	99,4%	100,0%	99,2%	100,0%	100,0%	100,0%	99,2%	98,6%	72,1%	98,0%
ScV-M2-2323 (OK412900)	99,8%	98,9%	100%	98,3%	98,9%	98,3%	98,3%	98,0%	98,3%	98,3%	98,3%	98,3%	98,6%	72,4%	98,0%
ScV-M2- QA23 (OK412901)	99,0%	99,9%	98,8%	100%	97,7%	99,4%	100,0%	99,2%	100,0%	100,0%	100,0%	99,2%	98,6%	72,1%	98,0%
ScV-M2-U43 (OK412902)	99,6%	98,7%	99,4%	98,6%	100%	97,7%	97,7%	97,5%	97,7%	97,7%	97,7%	97,7%	98,0%	71,8%	97,5%
ScV-M2- VL1(OK412903)	99,2%	99,2%	99,0%	99,1%	98,8%	100%	99,4%	99,2%	99,4%	99,4%	99,4%	99,2%	98,6%	72,1%	98,0%
ScV-M2- X5 (OK412904)	99,0%	99,9%	98,8%	99,8%	98,6%	99,1%	100%	99,2%	100,0%	100,0%	100,0%	99,2%	98,6%	72,1%	98,0%
ScV-M2- FX10 (OK412905)	99,3%	99,3%	99,1%	99,2%	98,9%	99,3%	99,2%	100%	99,2%	99,2%	99,2%	98,9%	98,3%	71,8%	97,7%
ScV-M2-VR5 (OK412906)	99,1%	100,0%	98,9%	99,9%	98,7%	99,2%	99,9%	99,3%	100%	100,0%	100,0%	99,2%	98,6%	72,1%	98,0%
ScV-M2- PDM (OK412907)	99,1%	100,0%	98,9%	99,9%	98,7%	99,2%	99,9%	99,3%	100,0%	100%	100,0%	99,2%	98,6%	72,1%	98,0%
ScV-M2- EZ44 (OK412908)	99,1%	100,0%	98,9%	99,9%	98,7%	99,2%	99,9%	99,3%	100,0%	100,0%	100%	99,2%	98,6%	72,1%	98,0%
K2 type killer virus (X54154)	99,3%	99,3%	99,1%	99,2%	98,9%	99,3%	99,2%	99,4%	99,3%	99,3%	99,3%	100%	98,3%	72,4%	97,7%
mRNA for K2 killer toxin (X56604)	99,4%	99,1%	99,3%	99,0%	99,1%	99,2%	99,0%	99,3%	99,1%	99,1%	99,1%	99,4%	100%	72,6%	98,3%
S.cerevisiae K-2 gene (X56987)	99,2%	98,9%	99,1%	98,8%	98,9%	99,0%	98,8%	99,1%	98,9%	98,9%	98,9%	99,2%	99,2%	100%	72,4%
ScV-M2-4 (MF957266)	98,7%	98,3%	98,5%	98,2%	98,3%	98,4%	98,2%	98,5%	98,3%	98,3%	98,3%	98,5%	98,7%	98,5%	100%
