# Supplementary material for: Identification and Molecular Characterization of Novel Mycoviruses in Saccharomyces and Non-Saccharomyces Yeasts of Oenological Interest
Source: Viruses. 2021 Dec 29;14(1):52. doi: 10.3390/v14010052 (PMC8778689; doi:10.3390/v14010052)
Supplement: Supplementary file 1 [file viruses-14-00052-s001.zip › viruses-1472465-supplementary/Supplementary Table S5.pdf]

Supplementary Table S5: Nucleotide percent identity (below diagonal) and amino acid percent identity (above diagonal) of ScV-L-BC isolates.

	ScV-L-BC-La-D254 (OK377018)	ScV-L-BC2-EC1118 (OK377019)	ScV-L-BC-La-RC212 (OK377020)	ScV-L-BC2-QA23 (OK377021)	ScV-L-BC2-X5 (OK377022)	ScV-L-BC2-VR5 (OK377023)	ScV-L-BC2-PDM (OK377024)	ScV-L-BC2-EZ44 (OK377025)	ScV-L-BC-lus (KT784813)	ScV-L-BC2 (KX906605)	ScV-L-BC-La (U01060)
ScV-L-BC-La-D254 (OK377018)	100%	97,4%	100,0%	97,4%	97,4%	97,4%	97,4%	97,7%	93,2%	97,4%	97,0%
ScV-L-BC2-EC1118 (OK377019)	95,5%	100%	97,4%	100,0%	99,6%	99,6%	99,6%	99,2%	94,0%	100,0%	97,0%
ScV-L-BC-La-RC212 (OK377020)	100,0%	95,5%	100%	97,4%	97,4%	97,4%	97,4%	97,7%	93,2%	97,4%	97,0%
ScV-L-BC2-QA23 (OK377021)	95,5%	100,0%	95,5%	100%	99,6%	99,6%	99,6%	99,2%	94,0%	100,0%	97,0%
ScV-L-BC2-X5 (OK377022)	95,5%	99,9%	95,5%	99,9%	100%	100,0%	100,0%	99,6%	94,0%	99,6%	97,4%
ScV-L-BC2-VR5 (OK377023)	95,5%	99,9%	95,5%	99,9%	100,0%	100%	100,0%	99,6%	94,0%	99,6%	97,4%
ScV-L-BC2-PDM (OK377024)	95,5%	99,9%	95,5%	99,9%	100,0%	100,0%	100%	99,6%	94,0%	99,6%	97,4%
ScV-L-BC2-EZ44 (OK377025)	95,6%	99,8%	95,6%	99,8%	99,9%	99,9%	99,9%	100%	93,6%	99,2%	97,7%
ScV-L-BC-lus (KT784813)	90,0%	90,0%	90,0%	90,0%	90,0%	90,0%	90,0%	89,9%	100%	94,0%	94,0%
ScV-L-BC2 (KX906605)	95,5%	100,0%	95,5%	100,0%	99,9%	99,9%	99,9%	99,8%	90,0%	100%	97,0%
ScV-L-BC-La (U01060)	96,1%	94,5%	96,1%	94,5%	94,6%	94,6%	94,6%	94,8%	89,3%	94,5%	100%
