# Supplementary material for: Identification and Molecular Characterization of Novel Mycoviruses in Saccharomyces and Non-Saccharomyces Yeasts of Oenological Interest
Source: Viruses. 2021 Dec 29;14(1):52. doi: 10.3390/v14010052 (PMC8778689; doi:10.3390/v14010052)
Supplement: Supplementary file 1 [file viruses-14-00052-s001.zip › viruses-1472465-supplementary/Supplementary Table S6.pdf]

Supplementary table S6: Amino acid percent identities of SbTV1 for Coat Protein gene.

	DCADSRV-7 (YP_009553337)	SbTV1 (OK412911)	RaTV (BBZ90081)	MoV2 (BCL64969)	MpV3 (QKO02073)
DCADSRV-7 (YP_009553337)	100%				
SbTV1 (OK412911)	30,2%	100%			
RaTV (BBZ90081)	31,0%	29,6%	100%		
MoV2 (BCL64969)	35,4%	40,3%	41,2%	100%	
MpV3 (QKO02073)	32,0%	37,0%	38,2%	67,3%	100%
