# Supplementary material for: Identification and Molecular Characterization of Novel Mycoviruses in Saccharomyces and Non-Saccharomyces Yeasts of Oenological Interest
Source: Viruses. 2021 Dec 29;14(1):52. doi: 10.3390/v14010052 (PMC8778689; doi:10.3390/v14010052)
Supplement: Supplementary file 1 [file viruses-14-00052-s001.zip › viruses-1472465-supplementary/Supplementary Table S7.pdf]

Supplementary Table S7: Amino acid percent identities of SbTV1 for RNA dependent RNA polymerase gene.

	LRV1 (AHJ90430)	LRV1 (ASK05972)	GARV-L1 (NP_624332	RaTV (BBZ90082)	SbTV1 (OK412911)
LRV1 (AHJ90430)	100%				
LRV1 (ASK05972)	89,6%	100%			
GARV-L1 (NP_624332)	36,1%	36,3%	100%		
RaTV (BBZ90082)	38,9%	39,2%	40,5%	100%	
SbTV1 (OK412911)	41,8%	41,3%	42,3%	43,9%	100%
