# Supplementary material for: Identification and Molecular Characterization of Novel Mycoviruses in Saccharomyces and Non-Saccharomyces Yeasts of Oenological Interest
Source: Viruses. 2021 Dec 29;14(1):52. doi: 10.3390/v14010052 (PMC8778689; doi:10.3390/v14010052)
Supplement: Supplementary file 1 [file viruses-14-00052-s001.zip › viruses-1472465-supplementary/Supplementary Table S8.pdf]

Supplementary Table S8: Amino acid percent identities of ScCV1 isolates for RNA dependent RNA polymerase.

	CSpV1 (BAU19315)	CSpV1 (BAU19321)	CSpV1 (BAU19324)	CSpV1 (BAU19329)	CSpV1 (BAU19313)	CSpV1 (BAU19327)	CSpV1 (BAU19320)	CSpV1 (BAU19316)	CSpV1 (BAU19333)	CSpV1 (BAU19330)	CSpV1 (BAU19332)	CSpV1 (BAU19314)	CSpV1 (QBA97381)	CSpV1 (BAU19331)	CSpV1 (BAU19319)	CSpV1 (BAU19335)	CSpV1 (BAU19317)	CSpV1 (ABV83023)	CSpV1 (AAC47806)	ScCV1-D254 (OK412915)	ScCV1-U43 (OK412916)
CSpV1 (BAU19315)	100%																				
CSpV1 (BAU19321)	98,9%	100%																			
CSpV1 (BAU19324)	98,5%	97,7%	100%																		
CSpV1 (BAU19329)	98,7%	97,9%	99,4%	100%																	
CSpV1 (BAU19313)	99,8%	99,0%	98,7%	98,9%	100%																
CSpV1 (BAU19327)	99,0%	98,7%	98,3%	98,5%	99,2%	100%															
CSpV1 (BAU19320)	99,6%	98,9%	98,9%	99,0%	99,8%	99,4%	100%														
CSpV1 (BAU19316)	99,6%	98,8%	99,0%	99,0%	99,8%	99,4%	100,0%	100%													
CSpV1 (BAU19333)	99,8%	99,0%	98,8%	98,8%	100,0%	99,2%	99,8%	99,8%	100%												
CSpV1 (BAU19330)	99,0%	98,2%	98,1%	98,1%	99,2%	98,8%	99,0%	99,0%	99,2%	100%											
CSpV1 (BAU19332)	99,4%	98,9%	98,3%	98,5%	99,6%	99,4%	99,4%	99,4%	99,6%	99,2%	100%										
CSpV1 (BAU19314)	99,6%	98,9%	98,5%	98,7%	99,8%	99,0%	99,6%	99,6%	99,8%	99,0%	99,4%	100%									
CSpV1 (QBA97381)	99,0%	98,7%	97,9%	98,1%	99,2%	98,5%	99,0%	99,0%	99,2%	98,4%	98,9%	99,0%	100%								
CSpV1 (BAU19331)	99,4%	98,8%	98,4%	98,4%	99,6%	99,4%	99,4%	99,4%	99,6%	99,2%	100,0%	99,4%	98,8%	100%							
CSpV1 (BAU19319)	99,4%	98,8%	98,2%	98,4%	99,6%	99,4%	99,4%	99,4%	99,6%	99,2%	100,0%	99,4%	98,8%	100,0%	100%						
CSpV1 (BAU19335)	99,0%	98,5%	97,9%	98,1%	99,2%	99,0%	99,0%	99,0%	99,2%	98,8%	99,6%	99,0%	98,5%	99,6%	99,6%	100%					
CSpV1 (BAU19317)	99,6%	98,8%	98,4%	98,6%	99,8%	99,0%	99,6%	99,6%	99,8%	99,0%	99,4%	99,6%	99,0%	99,4%	99,4%	99,0%	100%				
CSpV1 (ABV83023)	99,0%	98,3%	98,3%	98,5%	99,2%	98,9%	99,4%	99,4%	99,2%	98,4%	98,9%	99,0%	98,5%	98,8%	98,8%	98,5%	99,0%	100%			
CSpV1 (AAC47806)	98,3%	97,5%	97,1%	97,3%	98,5%	97,7%	98,3%	98,2%	98,4%	97,7%	98,1%	98,7%	97,7%	98,1%	98,0%	97,7%	98,3%	97,7%	100%		
ScCV1-D254 (OK412915)	35,9%	35,9%	35,7%	35,7%	35,7%	35,7%	35,7%	36,9%	36,2%	36,3%	35,7%	35,7%	35,7%	36,2%	37,0%	35,5%	36,0%	35,1%	35,7%	100%	
ScCV1-U43 (OK412916)	35,9%	35,9%	35,7%	35,7%	35,7%	35,7%	35,7%	36,9%	36,2%	36,3%	35,7%	35,7%	35,7%	36,2%	37,0%	35,5%	36,0%	35,1%	35,7%	99,4%	100%
