# Supplementary material for: Identification and Molecular Characterization of Novel Mycoviruses in Saccharomyces and Non-Saccharomyces Yeasts of Oenological Interest
Source: Viruses. 2021 Dec 29;14(1):52. doi: 10.3390/v14010052 (PMC8778689; doi:10.3390/v14010052)
Supplement: Supplementary file 1 [file viruses-14-00052-s001.zip › viruses-1472465-supplementary/Supplementary Table S9.pdf]

**Supplementary Table S9:** Amino acid percent identities of ScCV1 isolates for Coat protein.

| | CSpV1 (AAC47806.1) | CSpV1 (BAU19345) | CSpV1 (BAU19344) | CSpV1 (BAU19352) | CSpV1 (BAU19360) | CSpV1 (BAU19353) | CSpV1 (BAU19354) | CSpV1 (ABV83024) | CSpV1 (BAU19355) | CSpV1 QBA97382.1 | CSpV1 (BAU19364) | CSpV1 (BAU19365) | *C. hominis* (ABB02500.1) | *C. meleagridis* (ABB02501) | *C. felis* (ABB02502) | ScCV1-D254 (OK412913) | ScCV1-U43 (OK412914) |
|---|---|---|---|---|---|---|---|---|---|---|---|---|---|---|---|---|---|
| CSpV1 (AAC47806.1) | 100% | | | | | | | | | | | | | | | | |
| CSpV1 (BAU19345) | 99,7% | 100% | | | | | | | | | | | | | | | |
| CSpV1 (BAU19344) | 99,7% | 100,0% | 100% | | | | | | | | | | | | | | |
| CSpV1 (BAU19352) | 99,4% | 99,7% | 99,7% | 100% | | | | | | | | | | | | | |
| CSpV1 (BAU19360) | 99,7% | 100,0% | 100,0% | 99,7% | 100% | | | | | | | | | | | | |
| CSpV1 (BAU19353) | 99,4% | 100,0% | 100,0% | 99,4% | 100,0% | 100% | | | | | | | | | | | |
| CSpV1 (BAU19354) | 99,7% | 100,0% | 100,0% | 99,7% | 100,0% | 100,0% | 100% | | | | | | | | | | |
| CSpV1 (ABV83024) | 98,7% | 99,3% | 99,3% | 98,7% | 99,3% | 98,7% | 99,3% | 100% | | | | | | | | | |
| CSpV1 (BAU19355) | 99,1% | 100,0% | 100,0% | 99,1% | 99,7% | 99,1% | 99,7% | 98,7% | 100% | | | | | | | | |
| CSpV1 QBA97382.1 | 99,7% | 100,0% | 100,0% | 99,7% | 100,0% | 99,7% | 100,0% | 99,1% | 99,4% | 100% | | | | | | | |
| CSpV1 (BAU19364) | 99,0% | 99,3% | 99,7% | 99,0% | 99,3% | 99,3% | 99,3% | 98,7% | 99,3% | 99,3% | 100% | | | | | | |
| CSpV1 (BAU19365) | 99,3% | 99,7% | 99,7% | 99,3% | 99,7% | 99,7% | 99,7% | 99,0% | 99,7% | 99,7% | 100,0% | 100% | | | | | |
| *C. hominis* (ABB02500.1) | 93,1% | 93,4% | 93,3% | 92,8% | 93,4% | 92,8% | 93,4% | 92,2% | 92,5% | 93,1% | 92,7% | 93,0% | 100% | | | | |
| *C. meleagridis* (ABB02501) | 86,2% | 88,0% | 88,0% | 86,2% | 88,1% | 86,5% | 88,2% | 86,2% | 86,2% | 86,5% | 87,7% | 88,0% | 85,9% | 100% | | | |
| *C. felis* (ABB02502) | 88,7% | 90,0% | 90,0% | 88,7% | 90,1% | 89,0% | 90,2% | 88,1% | 88,4% | 89,0% | 89,7% | 90,0% | 87,5% | 91,2% | 100% | | |
| ScCV1-D254 (OK412913) | 20,5% | 21,2% | 21,2% | 20,2% | 21,1% | 20,5% | 20,9% | 20,2% | 20,8% | 20,5% | 21,2% | 21,3% | 21,2% | 21,8% | 21,2% | 100% | |
| ScCV1-U43 (OK412914) | 20,5% | 21,2% | 21,2% | 20,2% | 21,1% | 20,5% | 20,9% | 20,2% | 20,8% | 20,5% | 21,2% | 21,3% | 21,2% | 21,8% | 21,2% | 98,7% | 100% |
